# Multifactorial Contribution of Notch Signaling in Head and Neck Squamous Cell Carcinoma

**DOI:** 10.3390/ijms20061520

**Published:** 2019-03-26

**Authors:** Cristina Porcheri, Christian Thomas Meisel, Thimios Mitsiadis

**Affiliations:** University of Zurich, Institute of Oral Biology, Plattenstrasse 11, CH-8032 Zurich, Switzerland; cristina.porcheri@zzm.uzh.ch (C.P.); christian.meisel@zzm.uzh.ch (C.T.M.)

**Keywords:** Notch pathway, oral cancer, squamous cell carcinoma

## Abstract

Head and neck squamous cell carcinoma (HNSCC) defines a group of solid tumors originating from the mucosa of the upper aerodigestive tract, pharynx, larynx, mouth, and nasal cavity. It has a metastatic evolution and poor prognosis and is the sixth most common cancer in the world, with 600,000 new cases reported every year. HNSCC heterogeneity and complexity is reflected in a multistep progression, involving crosstalk between several molecular pathways. The Notch pathway is associated with major events supporting cancerogenic evolution: cell proliferation, self-renewal, angiogenesis, and preservation of a pro-oncogenic microenvironment. Additionally, Notch is pivotal in tumor development and plays a dual role acting as both oncogene and tumor suppressor. In this review, we summarize the role of the Notch pathway in HNSCC, with a special focus on its compelling role in major events of tumor initiation and growth.

## 1. Introduction

### 1.1. Developmental Stages of Oral Squamous Carcinoma

Solid tumors are abnormal cellular masses that originate from a cohort of cells within the tissue, disrupting its structure and organization. In dependence of the tissue of origin, solid tumors are classified into either sarcomas or carcinomas. Sarcomas originate from transformed cells of mesenchymal origin in the bone or the soft tissues (e.g., cartilage, muscles, vascular tissue, or connective tissue). Carcinomas arise from epithelial cells that line the wall of a variety of organs, such as the skin, the lungs, the digestive system, and the oral cavity. The most common types of carcinoma are basal cell carcinoma and squamous cell carcinoma. While basal cell carcinoma is the most common cancer of the skin, squamous cell carcinoma is the most common in head and neck cancer, accounting for approximately 90% of cases [1].

Head and Neck Squamous Cell Carcinoma (HNSCC) encompasses all tissues of the oral cavity. This cancer is often highly aggressive, especially in young patients under 40 years of age, and with a poor prognosis if diagnosed in advanced stages (III–IV), due to metastasis [2,3,4].

**Figure 1:**
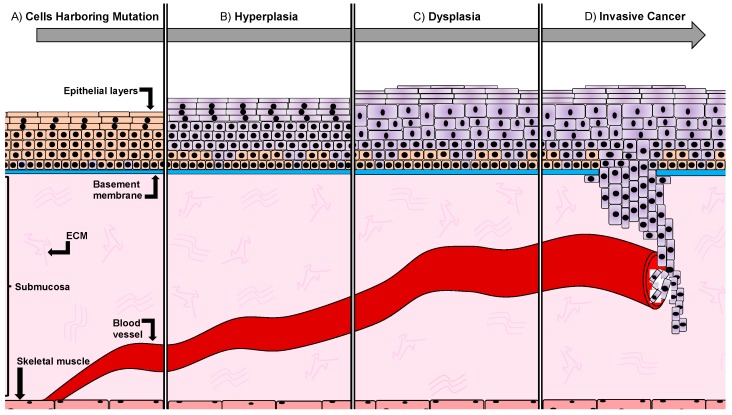
Developmental stages of squamous cell carcinoma. (**A**) The basement membrane (blue line) separates the epithelium and the mesenchyme/submucosa, which contains the extracellular matrix (ECM) (pink lines) and the blood vessel (red). Skeletal muscle cells are located beneath the submucosa (dark pink). Oral epithelium, starting above the basement membrane (blue), consists of several layers (orange). Cancer stem cells (purple) harboring a mutation are located in the first layer above the lamina propria. (**B**) Rapid and strong proliferation of epithelial cells (hyperplasia) leads to a thickening of the epithelium. Cells carrying mutations are shown in purple. (**C**) Proliferating epithelial cells change morphology but remain locally confined in the epithelial layer. Hyperkeratosis is detectable in the most external epithelial layer. (**D**) Cancer cells breach the basement membrane, invading the underlying mesenchyme and neighboring tissues. Invasive cancer cells enter the blood stream and establish new secondary tumors (metastases) in distant organs.

Histologically, the carcinogenesis is divided into three progressive stages: hyperplasia, atypical hyperplasia (dysplasia), and invasive cancer (Figure 1).

Hyperproliferation leads to an increase in cell numbers of the epithelium (hyperplasia), without a change in shape. If cell division becomes deregulated, cells become abnormal in shape and lose classical morphological features, a stage called dysplasia. As a result, the tissue has a disordered appearance and changes in cell morphology can be observed. However, dysplasia is not a guarantee that cells have acquired a cancerous stage, though possible genetic alterations have occurred leading to a predisposition of cancer.

In a higher grade of dysplasia, the carcinoma in situ, epithelial cells remain within their tissue without spreading into adjacent structures. At advanced stages, dysplastic cells breach the basement membrane and invade nearby tissues and organs, forming metastases (Figure 1).

**Figure 2:**
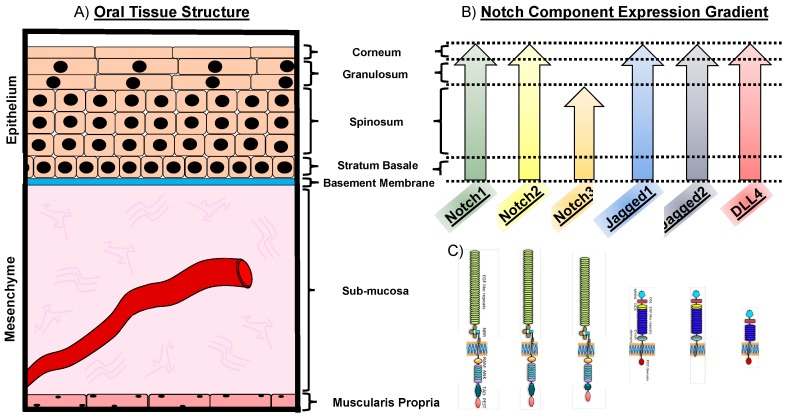
Notch expression in oral structures. (**A**) Oral epithelium consists of the stratum basale, spinosum, granulosum and corneum. The mesenchymal compartment beneath the epithelium contains the submucosa, ECM and blood vessels. (**B**) Expression of Notch signaling components within the oral mucosa. Notch1, Notch2, Jagged1, Jagged2, and DLL4 are expressed throughout the epithelial structures. The Notch3 receptor expression is limited to the layers of stratum basale and spinosum. (**C**) Schematic representation of the molecular structure of receptors Notch1,2,3 and the ligands Jagged1,2 and DLL4. Dotted lines represent the expression of the Notch signaling components in the corresponding epithelial layer.

Oral cancer occurs in several oral structures, of which the tongue, the salivary glands and the mucosa lining the inner wall of mouth and the nasal cavity are the most commonly affected [5]. Intraorally, 40–50% of oral cancers commonly affect the tongue [6]. The lingual epithelial tissue is thought to be the origin of squamous cell carcinoma of the tongue. The epithelium of the tongue consists of four tightly packed layers, with a varying degree of differentiation. The basement membrane marks the histological border between the stratified epithelia and the mesenchymal compartment. Cells of the basal layer are relatively round in shape and proliferative. The ratio of nuclear versus cytoplasmic size is reduced the further the cells differentiate and migrate toward the outer layer, while they additionally flatten out and become squamous in shape. Above the submucosa, which contains blood vessels, minor salivary glands, nerves, structural fibers, fibroblasts, and other cell types, lies the stratum basale (basal layer) (Figure 1 and Figure 2A). It consists of a layer of cuboidal cells and it is thought to harbor the undifferentiated cells of the tongue epithelium. These cells have organelles which are typical for protein-producing cells and express intermediate filaments of cytokeratin 5 and 14, respectively. Basal cells divide continuously and differentiate toward the outer layers. The stratum spinosum (prickle layer) contains cells that are characteristically bigger in volume. These cells become more squamous shaped and produce differentiation-specific keratins e.g., 1, 6, 10, and 16 (Figure 1 and Figure 2A). The granular layer is characteristically built-up by flattened cells and contains the basophilic keratohyalin granules and densely packed keratin filaments. The outmost layer is the stratum corneum, consisting of a pure keratinized layer. These cells have no organelles and no more nuclei are present (orthokeratinized) (Figure 1 and Figure 2A). In HNSCC, the epithelial layer is thought to be the affected tissue, leading to cancer formation. In the early stage of oral cancer, cells undergo hyperproliferation, increasing their number in the lower third of the epithelium, but display a normal differentiation program [7]. In advanced cancer stages, squamous cells become dysplastic, i.e., highly proliferative and morphologically abnormal, starting to infiltrate different layers of the epithelium [8] (Figure 1 and Figure 2A). Ultimately, these cells degrade the subepithelial basement membrane and invade the underlying tissue structure, thus acquiring the ability to invade distant tissues and form metastases (Figure 1).

Salivary gland tumors account for up to 6.5% of all head and neck cancers [9]. The salivary glands comprise the parotid, submandibular, and sublingual glands, together with minor glands scattered over the labial, buccal, palatal, and lingual surfaces of the mouth cavity [10]. Tumors can originate in either the major or the minor salivary glands, but approximately 80% of them arise in the parotid gland, 15% in the submandibular gland, and 5% in the sublingual and minor glands (2005 World Health Organization (WHO) classification of epithelial salivary gland tumors). Salivary glands are divided into lobules by connective tissue septa engulfing the functional portion of secretory units (acini) and ducts. Acini can be serous, if they secrete proteins dissolved in isotonic water fluid; mucous, if they secrete the lubricant mucous or mixed, histologically distinguishable for their demilune structure, where serous acini surround mucous acini [11]. The secretory units abut onto the intercalated ducts, limited by a row of cuboidal epithelium and myoepithelial cells underneath. These ducts continue as striated ducts, with a basement membrane to allow water reabsorption and ion secretion. The striated ducts end into interlobular ducts, enclosed by columnar epithelium [10]. More than 95% of salivary gland tumors are of epithelial origin, with the most malignant form being represented by the basal cell adenocarcinoma, mucoepidermoid carcinoma, adenoid cystic carcinoma, and squamous cell carcinoma. Due to the limited molecular understanding of the salivary gland tumors, the majority of patients undergo a surgical removal of the primary tumor followed by radiotherapy. Surgical intervention has severe limitations in efficiency, and recurrence occurs in 40–45% of cases as a consequence of incomplete resection. Additionally, the invasive nature of the procedure often leads to serious side effects, such as debilitating facial disfigurement. Surgery is normally accompanied by radiotherapy, that directly affects salivary glands functionality, leading to dryness, increased risk of oral infections, problems in mastication, and impaired language skills [12,13,14].

HNSCC encompasses many site-specific oropharyngeal cancers [15]. Typical risk factors for these oral epithelial cancers can be subdivided into three groups: chronic irritants of the aerodigestive tract (e.g., tobacco and alcoholic beverages), genetic syndromes caused by genomic instability and DNA repair defects, and human papillomavirus infections leading to cell cycle alterations. Patients with a history of tobacco consumption have more gene mutations associated with tumor growth than non-tobacco users, especially when combined with the abuse of alcohol [16]. Additionally, a diet low in fruits and vegetables has been connected to favor the development of oral squamous cell carcinoma [14,16,17,18].

The most conventional methods of treatment are limited to surgical removal of the primary tumor, radiation, and chemotherapy. Treatment of early HNSCC usually involves either radiation or surgery, with a favorable prognosis [19].

However, currently the 5-year overall survival rate after conventional treatment is approximately at 40–50%, due to high incidence of treatment resistance, consequent recurrence of the tumor, and following lymph node metastasis [20].

Therefore, to develop efficient and ultimate treatment therapies, it is paramount to identify cellular and molecular mechanisms of HNSCC tumor formation, progression, treatment resistance and metastasis. Normal epithelial cells might develop into cancerous cells as a consequence of specific genetic alterations, gene deletions/amplifications or epigenetic modifications. Often these genes are key players in regulating cell cycle and proliferation such as the Notch, Wingless/Integrated (Wnt), Nuclear factor kappa-light-chain-enhancer of activated B cells (NF-κB), p53, or mitogen-activated protein kinase (MAPK) pathways.

### 1.2. The Notch Pathway in Oral Physiology

The Notch pathway is an evolutionary well conserved pathway involved in cell-to-cell communication [21]. It is required for cell fate decisions at multiple stages of embryonic development as well as in the adult organism, while dysregulation of the pathway is associated with genetic and acquired diseases, including cancer.

The activation of the Notch pathway is initiated when a membrane-bound Notch receptor interacts with a specific ligand on the adjacent cell, therefore regulating intercellular signaling. In mammals, four Notch receptors have been described (Notch1, Notch2, Notch3, and Notch4) and five ligands: two of the Jagged family (Jagged 1 and Jagged2) and three members of the Delta-like family (Dll1, Dll3, Dll4). The canonical Notch signaling pathway is initiated by the interaction between ligand and Notch receptor on a juxtaposed cell in trans. Upon ligand association, the ligand is endocytosed, leading to a pulling force on the Notch Extracellular Domain (NECD) of the receptor. As a result, the three cysteine-rich Lin-12/Notch Repeats (LNR) are exposing the S2 cleavage site on the receptor [21,22,23]. The S2 cleavage site is proteolytically processed by A Disintegrin And Metalloproteinase 10/17 (ADAM10/ADAM17) [22]. Upon S2 cleavage, the membrane-tethered Notch extracellular truncation (NEXT) is formed. NEXT harbors the substrate for the γ-secretase complex. The γ-secretase complex cleaves NEXT within the transmembrane domain at the S3 cleavage site. This creates a Notch intracellular domain (NICD). Upon S3 cleavage, the NICD binds to importin α3, α4, or α7 [24] with its nuclear localizing sequence (NLS). In the canonical model, NICD interacts with recombining binding protein suppressor of hairless (RBPj) (alternatively known as: CSL, C promoter-binding factor 1 (CBF1)/Suppressor of Hairless/Lag-1), and translocates into the nucleus to regulate the transcription of specific Notch-target genes (such as Hairy And Enhancer Of Split 1 (HES)1, HES5, Hairy/enhancer-of-split related with YRPW motif 1 (HEY)). In the noncanonical activation of the pathway, Notch cleavage is also induced by ligand–receptor interaction on the surface, but pathway transduction works independently from RBPj. Recently, noncanonical activation of Notch has been associated with tumorigenic events in various cancers (breast cancer tumor progression, leukemia and hematopoietic proliferation, neuroblastoma models) [25,26,27,28,29,30]. Post-translational modification of the Notch receptors and ligands can influence their level of activation, which subsequently affects downstream targets. One of these processes is glycosylation. The level of glycosylation of the Notch receptor directly influences the affinity of the receptor for a specific ligand. A specialized family of N-acetylglucosaminidyltransferases, the Fringe enzymes, initiate the elongation of the O-linked fucose residues on the epidermal growth factor (EGF)-like repeats, preventing interaction of Notch with Jagged ligands, but not Delta-like ligands. Initially identified in Drosophila, three homologues of the Fringe proteins have been identified in mammals: Lunatic Fringe (Lfng), Maniac Fringe (Mnfg), and Radical Fringe (Rfng) [31,32,33]. They play an essential role during embryonic development, as demonstrated by somites malformation in the *Lfng*-mutant. Recently, their importance has been associated with cancer development, where decreased levels of *Lfng* are a hallmark of triple-negative breast cancer and *Lfng* blocked mammary stem cell proliferation [34]. In conclusion, the Notch pathway can be regulated at various levels, besides the mere control of genetic expression. Its duration and timing of activation might therefore largely vary due to the extracellular conditions to which the cell is exposed. These factors highlight the importance of studying the pathway within its tissue context, maintaining the complexity of the surrounding microenvironment.

In the oral cavity, members of the Notch pathway are mainly confined to the oral mucosa. The oral mucosa represents the biggest organ of the oral cavity containing temperature and tactile receptors and can be subdivided into three types: (i) The lining mucosa is the most represented in the oral tissue covering 60% of the surface area, (ii) the masticatory mucosa (representing approximately 25%), and (iii) the specialized mucosa (15% of the total oral mucosa) [35]. The lining mucosa is a stratified squamous nonkeratinized epithelium supported by a more elastic and flexible connective tissue. This mucosa type lines the surface of the lips, cheeks, floor of the mouth and covers the ventral area of the tongue. The masticatory mucosa represents a keratinized epithelium and is tightly attached to the underlying tissues by a collagenous connective tissue, or lamina propria. This mucosa is designated to withstand abrasion due to mastication and covers tissues such as the gums and the palate. The specialized mucosa lines the dorsal part of the tongue. It is a masticatory mucosa by function, but additionally characterized by its high extensibility and lingual papillae. Notch1 expression is detectable throughout all mucosa types, although with varying degree of intensity within the epithelial layers, i.e., higher expression is detectable in the stratum basale and spinosum, while it is faintly expressed in the stratum granulosum and corneum [36,37,38]. Notch2 receptor is expressed in the tongue squamous epithelium, [39,40], whereas Notch3 is expressed in the stratum basale and spinosum [35,38,39] (Figure 2). The ligand Jagged1 was reported to be strongly expressed in the epithelial layers stratum basale and spinosum, while a gradually fainting signal was detected in the outer layers stratum granulosum and corneum [36,41]. Jagged2 expression was detected throughout the epithelial layers of the tongue, resembling the expression pattern of Notch1. However, a strong expression of Jagged2 limited to the stratum basale was also reported [35,38,39]. Throughout the epithelial oral mucosa layers, only a low expression was reported for the ligand DLL4 [38,39] (Figure 2).

To support oral homeostasis and functionality, secretion from the salivary glands helps preserving a healthy oral environment, and it is essential for mastication and speech.

The Notch signaling pathway is expressed in submandibular gland tissue, although its role has not been fully characterized. Notch1-4 receptors are present in the normal salivary gland tissue, as well as the ligands Jagged1, 2, and Delta1 (DLL1) [42]. Expression was found scattered in the ductal as well as acinar cells of the tissue, of which the latter often displayed a nuclear staining.

In conclusion, components of the Notch signaling pathway are present in the major structures of the oral cavity and potentially partake in their functionality.

### 1.3. Notch in Oral Pathological Conditions

Mutations in the Notch pathway lead to a variety of disorders and malformations. Craniofacial disorders, such as cleft lips and palate represent the most common developmental defects in humans, and also depends on an aberrant reorganization of the epithelial layer during palate elevation and fusion. The interaction Notch-Jagged has been directly associated with misregulated fusion, and mutant mouse models for Jagged2 develop palate clefting [43,44].

Alagille syndrome is a genetic disorder characterized by a number of abnormalities, which include ocular abnormalities, heart defects (pulmonic stenosis; ventricular septal defect), vertebral malformations, characteristic facial features, and cholestasis. Based on genetic screenings, most cases are thought to be caused by mutations in the Jagged1 and Notch2 genes [45].

In teeth, the Notch pathway plays a crucial role in the development of tooth germ and it is involved in regeneration of injured tissue in the adult teeth. Notch is essential for odontoblasts differentiation, mineralization of hard tissue, determination of the cusp architecture, and root formation. Upon carious or traumatic injury, the Notch signaling is triggered in pulpal mesenchymal cells, suggesting a role of the pathway in repair [42,46,47,48,49,50].

During vasculature establishment and maintenance, the altered expression of Notch3 and Notch4 receptors results in arteriovenous malformation. Sporadic lesions might occur that lead to increased blood flow and high pressure, disrupting the integrity of vessels and exposing them to facilitated rupture [51].

In important muscular diseases, such as the Duchenne Muscular Dystrophy, the role of Notch in maintenance of tissue homeostasis is linked with its involvement in stem cell-dependent regeneration [52,53]. Oral muscles are progressively affected by the disease, and potential restoration of their functionality might derive from the reduction in Jagged1 expression [54,55]. Finally, during thyroid development, malformation can occur to generate ectopic portion of thyroid tissue located at the base of the tongue. The structure takes the name of lingual thyroid and its origin is likely controlled by the Notch signal normally regulating correct thyroid formation via the Jagged–Notch axis [43,56,57].

### 1.4. Notch Expression in Oral Squamous Cell Carcinoma

Expression of Notch1 is pivotal for early cancer development. Similarly to other carcinoma, such as skin squamous cell carcinoma, the expression of Notch1 in the oral squamous epithelium was localized in the basal cells, which was found to be significantly downregulated in oral epithelial dysplasia [58]. Loss of Notch1 promotes a tumor-inducing effect, impairing barrier integrity and generating a wound-like environment in the underlying stroma. These findings strongly suggest that a reduction in Notch1 activity is a crucial event in oral cancer formation and progression. Based on bioinformatic studies and evaluations of Oral Squamous Cell Carcinoma (OSCC) data sets, the receptor Notch1 was the 4th highest protein of interest involved in oral cancer [59]. Inactivating mutations of Notch1 can be found in approximately 10% of all cases of squamous cell carcinoma including the oral cavity [60], indicating that Notch1 is one of the most mutated gene in squamous cell carcinoma. In line with this data, Notch effectors Hes1 and Hey1 were overexpressed in 32% of HNSCC cases, but only in absence of Notch1 inactivating mutation [61].

Numb is a membrane-associated protein which is expressed inversely to Notch. Numb is able to function as a negative regulator of Notch by recruiting the ubiquitination machinery to the plasma membrane, leading to Notch receptor ubiquitination and its subsequent degradation [62,63,64]. Overexpression of Numb was identified in OSCC samples, although these results are in contradiction to previous findings in esophageal squamous cell carcinoma, where Numb transcripts were decreased [65]. Furthermore, in human OSCC cell lines the overexpression of Numb results in a decreased cell proliferation, migration, and invasion, which was also detectable in esophageal squamous cell carcinoma [65,66]. siRNA-treated cells in vitro, targeting Numb, resulted in increased cell growth as well as invasion. However, the in vivo translation of the analyses was not conclusive [66].

Jagged1 and Jagged2 transcripts were significantly increased in OSCC cell lines as well as patient samples in comparison to non-neoplastic tissue [36,67]. The ligand Jagged1 was shown to be upregulated in OSCC cell lines and tissue samples; interestingly the downregulation of Jagged1 in the OSCC resulted in a decrease of cell proliferation in vitro as well as tumor growth in vivo, identifying Jagged 1 and Jagged2 ligands as potential therapeutic targets [41,67].

The metalloprotease a disintegrin and metalloprotease domain 17 (ADAM17) is overexpressed in a human OSCC cell line leading to an increase in cell viability and migration. Furthermore, after the transplantation of ADAM17 overexpressing cells, an increase in tumor cell proliferation as well as in tumor size could be detected [68]. In reverse, an inhibition of ADAM17 in HNSCC in vivo resulted in decreased tumorigenesis.

### 1.5. Notch and the Influence on Vasculature

**Figure 3:**
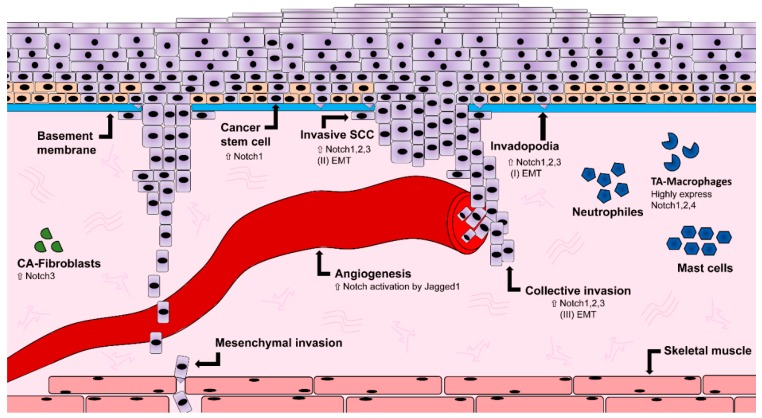
Notch is involved in structural alteration of the oral tissue. In the invasive cancer stage, cancer cells breach the basement membrane and invade the underlying mesenchyme to enter the blood stream. Cancer stem cells express Notch1. Invading cancer cells express Notch1, 2, and 3 undergoing epithelial–mesenchymal transition (EMT). Angiogenesis is triggered by Jagged1-dependent Notch activation. ECM-remodeling cancer-associated fibroblasts (CAFs) express Notch3, while tumor-associated macrophages (TA-Macrophages) highly express Notch1, 2, and 4.

A hallmark of cancer is increased angiogenesis. This process involves branching and formation of new blood vessels, required to guarantee optimal supply of nutrients for the tumor microenvironment. During angiogenic sprouting, tip endothelial cells start to migrate and proliferate in response to proangiogenic factors locally released. In contrast, neighboring stalk cells exposed to the same stimuli are resistant to migration and proliferation, remaining attached to the original vessels. The mechanism regulating endothelial sprouting is Notch-dependent in a variety of models (from zebrafish to mammals [69,70,71,72]). Connector and patent endothelial cells express high Notch, resulting in downregulation of the Vascular Endothelial Growth Factor (VEGF) transcript and its receptor VEGFR2 (also known as Kdr), which in turn controls endothelial migration and proliferation rate [73]. Consistently, tip endothelial cells have high level of VEGF and express the Dll4 ligand, able to activate Notch in the neighboring cell. On the other hand, interaction of the receptor with Jagged1 leads to reduced sprouting, suggesting that the choice of the ligands determine the balance between tip and stalk cells in arterial growth [74]. The Fringe glycosyltransferases, are central in this decision, as the interaction Notch-Dll4 is preferred over Notch-Jagged1 upon glycosylation of the ligand [74]. Tumor vessels abide to similar mechanisms of growth, using the Notch signaling to regulate the supply of blood borne factors. Pathological angiogenesis relies on local clues to recess or initiate growth. When a tissue lacks oxygen, blood vessels branch up into the hypoxic tissue to contrast tissue decay, and vasculature start to recess when vascular coverage allows sufficient perfusion. In contrast, tumor microenvironment continuously supports angiogenesis, due to the release of local inflammatory cytokines and angiogenic factors (Figure 3). Macrophages found in the tumor microenvironment participate in the regulation of vasculature remodeling, and strongly express Notch1, Notch2, and Notch4, together with VEGFR1 [75,76] (Figure 3). Additionally, tumor vasculature overexpresses the Dll4 ligand, resulting in a hypersprouting phenotype [77]. In lung cancer, VEGF directly affects expression of Dll4 in tumor vessels [78], as well as in neuroblastoma models, where blocking VEGFR2 increases the level of Jagged1 expression and consequent Notch1 hyperactivation [79]. Similarly to other solid tumors, HNSCC relies on the local network of blood vessels to support its growth, maintenance and metastatic invasion [80] (Figure 3). Particularly, the Notch ligand Jagged1 induces endothelial activation of Notch in HNSCC, promoting local sprouting [81]. The Notch3 receptor was reported to actively signal in stromal fibroblasts of OSCC human samples, inducing an increase in tumor angiogenesis [82]. In a HNSCC heterotopic xenograft tumor model, treatment with the epidermal growth factor receptor (EGFR) blocker Cetuximab inhibits angiogenesis by downregulating Notch1 and Hypoxia-inducible factor 1 alpha (Hif1α) subunit [83]. Finally, blocking Notch in tumor vasculature significantly reduces tumor growth, suggesting that endothelial Notch is a potential target for disrupting tumor microenvironment and pathological progression.

Summarizing, Notch signaling has a pivotal influence on regulating angiogenesis in various phases of oral tumor development, influencing its growth and formation of metastasis.

### 1.6. Notch and Epithelial-to-Mesenchymal Transition

Fate change is essential in tissue determination during embryonic development [84]. During gastrulation and neural crest stem cells’ migration, cells of epithelial origin face dramatic morphological changes, downregulate anchoring molecules, and increase their motility. Epithelial cells can be converted into fibroblast-like cells by changes in gene expression and cytoskeletal organization, in the process of Epithelial-to-Mesenchymal Transition (EMT). Thus, they can leave the ectoderm to migrate through the disrupted basal lamina underneath and form new distal organs. Once they have reached their destination, the process can be reverted, and mesenchymal cells might convert back into epithelial cells. Beside the process of organ formation during development, EMT occurs in repair processes, such as wound healing [85]. Dysregulation of EMT can result in scar formation and fibrosis with consequent malfunctioning of the organ [86]. Similarly, during metastases, cells change their structure and behavior activating the EMT program. They can invade the adjacent underlying tissues and form metastases [87]. At the molecular basis of EMT, dynamic remodeling in cadherin and integrin expression reflects in structural, motility, and fate changes of epithelial cells [88]. Notably, integrin β4, α5β1, and αVβ6 are among the first molecules being altered during metastasis, invasion, and progression of carcinoma cells. Additionally, changes in the cadherins panel of expression result in dramatic structural changes in epithelium, and specifically in the adherens junctions deprived of E-cadherin expression [89,90]. Cadherin switching from E-cadherin to N-cadherin is common during tumorigenic EMT, and correlates with increased migration, invasion, and poor prognosis in cancer. The transcription factor Snail1 is a major regulator of E-cadherin expression, reducing cell-to-cell adhesion and consequently causing destabilization of epithelial architecture and EMT. Notch1 overexpression leads to increased levels of the transcription factor Snail homolog 1. The use of gamma-secretase inhibitors N-[N-(3,5-difluorophenacetyl)-l-alanyl]-S-phenylglycine t-butyl ester (GSI DAPT) to inhibit Notch signaling in carcinoma, leads to an attenuation of transforming growth factor-β (TGF-β), which in turn controls Snail1 expression, indicating a role of Notch in EMT during cancer progression [91]. In a similar manner, the expression of receptors Notch2 and Notch3 correlates with Snail1 expression in cancer samples from patients with carcinoma of unknown primary (CUP syndrome) [92].

In a subset of lung cancer cells, Gefitinib-resistant cells displayed an EMT phenotype as well as an increase in Notch1 expression, when compared to their parental cells. On the other hand, when Notch1 was ablated in parental lung carcinoma cells, EMT was inhibited [93]. Gefitinib-sensitive parental cells were capable of acquiring an EMT phenotype upon Notch1 overexpression, which leads to the conclusion that Notch1 is a key player in the regulation of EMT [93]. In OSCC, it was shown that the expression of Notch receptors, ligands as well as Snail1 and other classical targets of Notch, are increased in hypoxic conditions. Hypoxia also decreased the expression of E-cadherin, leading to an increased motility of OSCC cell lines, an effect that could be stopped by inhibiting Notch signaling using GSI DAPT. This suggests that Notch might regulate EMT in OSCC cell lines under hypoxic conditions [94]. Cadherin switching was found in 30 of 80 HNSCC cases and correlates with histological changes, and lymph nodes metastasis [95]. Downregulation of E-cadherin has been found in OSCC cell lines, together with up-regulation of vimentin, a marker of mesenchymal phenotype [95]. Particularly, vimentin expression was localized in the cytoplasm of OSCC cells, directly at the invasive tumor front [95]. Taken together, these observations point at the Notch pathway as major regulator of EMT in a variety of cancerogenic conditions (Figure 3).

### 1.7. Notch and Cancer Stem Cells

Notch is crucial in stem cell maintenance and tissue regeneration processes [96]. Furthermore, a role for Notch in cancer stem cells have been hypothesized in the last years [97]. Investigations in breast cancer, glioma, pancreatic, prostate and hepatocellular carcinomas, have been linked to Notch and provided an insight to its direct influence on the maintenance of cancer stem cell (CSC) within the tumor [98,99,100,101,102,103,104]. CSC are able to self-renew and therefore take part in the regeneration of the tissue. A protective microenvironment preserves this ability, sustaining different signaling pathways, molecular circuits and epigenetic modifications.

A typical feature of the cancer stem cell phenotype is the resistance to antitumor treatments, such as radiation or chemotherapy, leading to a high incidence of reoccurrence of cancer in patients [97]. In breast cancer, Notch was found increased in stem cells derived mammospheres, suggesting an activation of the pathway in the subpopulation of self-renewing, undifferentiated progenitors [105]. Additionally, the use of GSI DAPT gamma-secretase inhibitor, strongly hampered sphere formation as well as their proliferation ability. Using a Her2/Neu positive xenograft model treated with GSI DAPT and Herceptin (a monoclonal antibody used in combination with chemotherapy), blocked cancer growth when compared with treatments with Herceptin alone and additionally prevent reoccurrence of the tumor [106]. Therefore, the use of GSI DAPT on some solid tumors indicates that Notch has a pivotal role in the maintenance of cancer stem cell-like cells.

Cancer stem cells have been identified in oral squamous cell carcinoma. Tumors generated from CD44-cancer stem cells sorted cells showed increased expression of β-catenin, E-cadherin, and low levels of Bmi1, Snail1, and Slug, all markers for increased tumorigenicity [107]. On the other hand, the usage of CD44 as CSC marker remains controversial, with some work demonstrating its expression in more differentiated cells [108]. Markers for undifferentiated progenitors can also be found in OSCC, such as octamer-binding transcription factor 4 (OCT4), NANOG, and sex determining region Y-box 2 (SOX2) [109,110] in association with oncogenes such as signal transducer and activator of transcription 3 (STAT3) (promoting pro-oncogenic inflammation) [111], CD24 (with angiogenic potential) [112], CD133, and Musashi1 (classically associated with undifferentiated, stem cell phenotype) [113,114]. In OSCC, the prolonged inflammatory microenvironment exposes CSCs to a continuous high level of tumor necrosis factor alpha (TNF-α), enhancing expression of genes for stem cells, chemo-resistance and the ability to produce tumoroids. In this context, Notch1 plays a coordinating role, as inhibition of the Notch-Hes1 signaling inhibits CSC phenotype in OSCC [115] (Figure 3).

### 1.8. The Dual Role of Notch as an Oncogene and a Tumor-Suppressor

The role of the Notch pathway in solid tumors remains controversial, as it has been associated with both tumorigenic and tumor-suppressive roles [116].

The neoplastic role of Notch was first discovered in patients suffering from human T cell acute lymphoblastic leukemia (T-ALL). In more than 50% of T-ALL cases, the patients have a chromosomal translocation (q34; q34.3) resulting in a truncated Notch receptor, which leads to a constantly active Notch1. Over the years, studies on the role of Notch in regulating the immune system unraveled its implication in inflammation and immune cell differentiation. The possibility to regulate the immune system is of great importance in tumorigenesis, as extrinsic and intrinsic inflammation leads to favorable conditions for tumor development. Notch functions in innate as well as adaptive immunity, by controlling the differentiation of dendritic cells (DCs), natural killer cells (NKs), and T cells (Th1, Th2, and Tregs). In physiological conditions, Notch is essential for T cell lineage commitment, where it acts as a checkpoint to guarantee T cell lineage differentiation by preventing the commitment to other lineages such as myeloid cells, DCs, and B cells [117,118]. Notch1 orchestrates the early phase of T-cell differentiation via DLL4 in the thymus, which requires a downregulation of expression for a full T-cell differentiation [119].

Opposing roles of Notch in regard to the control of cell fate decisions were reported for two kinds of NK cells at different maturation stages. Notch coordinates the differentiation of conventional NK cells, while the innate lymphoid cell (ILC)-derived natural cytotoxicity receptor (NCR) NKp44^+^ group (NCR^+^ILC3) remains suppressed in its differentiation [120].

**Figure 4:**
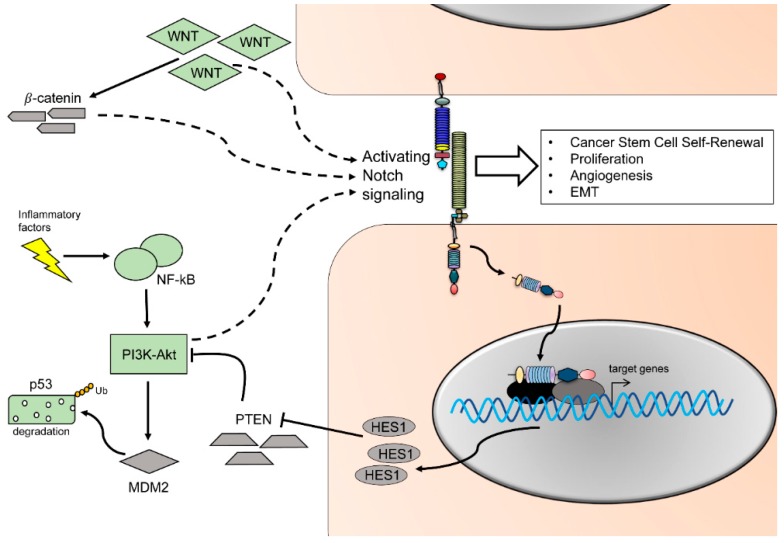
Molecular signaling in cancer. Crosstalk of Notch with other pathways influences cellular responses including cancer stem cell self-renewal, proliferation, angiogenesis, and EMT. Wnt signaling leads to β-catenin accumulation, which subsequently leads to an activation of Notch signaling and target gene transcription. Alternatively, aberrant Wnt signaling bypasses β-catenin, ultimately modulating Notch signaling. Inflammatory factors lead to an increase in NF-kB, which activates PI3K-Akt signaling which in turn triggers p53 ubiquitination and degradation. The expression of HES1 downregulates PTEN, leading to an increase in PI3K-Akt signaling, ultimately activating Notch signaling. Dotted lines indicate Notch pathway regulation. Black arrows illustrate activation of the pathway, T-arrows inhibition of the pathway.

Transient activity of Notch mediates myeloid differentiation by increasing mRNA levels of myeloid specific transcription factor PU.1 [121]. Notch ligands DLL and Jagged might lead to opposite effects on myeloid cells: DLL1-expressing fibroblasts promote DC differentiation and activation of Notch, while Jagged1 promotes immature myeloid cells [122]. It was reported that some signaling molecules involved in tumor-promotion, are modulated by the cells of the immune system, and might directly regulate the Notch pathway. High expression of Notch1, 2, and Jagged1 can be correlated with tumor progression of myeloma and it was proposed that Notch has an activating role of interleukin 6 (IL-6) proliferating signals in the bone marrow, enhancing tumor growth [123]. The cross-talk of TNF-*α*, Notch and inhibitor of nuclear factor kappa-B kinase subunit kinase β (Ikk2) (component of nuclear factor kappa-light-chain-enhancer of activated B cells (NF-kB) signaling pathway) leads to a suppression of the nuclear receptor Pparg, in an animal model of pancreatic cancer. Under normal conditions, Pparg encodes anti-inflammatory receptor Pparg. Hes1, a downstream target of the Notch pathway, inhibits Pparg which subsequently hampers the autocrine inflammatory activity of pancreatic tumor cells. As a result, production of inflammatory mediators (such as IL-6, IL-1*β*, and TNF-α) was enhanced, supporting inflammation and cancer progression via Notch pathway activation [124]. In OSCC cancer stem cells, NF-kB expression is specifically upregulated in both OSCC biopsies and orospheres self-renewal assays [125] (Figure 3 and Figure 4). In tongue squamous cell carcinomas, IL-1β upregulates CXC chemokine receptor 4 (CXCR4), leading to cancer growth and metastasis. This effect could be reversed by pharmacological interference of Notch1 signaling [126]. In conclusion, pro-inflammatory molecules such as IL-6, IL-8, and TNF-α can lead to Notch signaling activation, enhancing tumor-promoting effects on epithelial cells.

In several solid tumors, including OSCC, the tumor microenvironment plays an essential role in sustaining, nourishing, and protecting the cancerogenic core. The Notch signaling is implicated in the maintenance of the tumorigenic microenvironment from various fronts. The tumor stroma develops in parallel with the tumor epithelia, and it is involved in each step of cancer onset, development, and progression. This cooperation between epithelium and underlying mesenchyme often remains ignored, as many pharmacological treatments only target epithelium, neglecting the highly influential cancer stroma. The cancer-associated fibroblasts (CAFs), build the majority of the cancer stroma in a number of different cancers. CAFs directly influence the tumor microenvironment, secreting chemokines, cytokines, and growth factors, and play a role in the degradation of the extracellular matrix [127]. These critical features make CAFs a strong protumorigenic factor. Recent studies report that CAFs are capable of secreting a significantly higher amount of diffusible H_2_O_2_ than normal fibroblast, thereby acting as a field effect carcinogen [128,129]. This leads to a protumorigenic stromal environment, by increasing inflammatory and mitogenic factors, favoring primary epithelial cell transformation, and increasing cancer cell aggressiveness [129]. Elevated levels of the Notch1 receptor have been found during CAF activation, as shown in melanoma, where Notch1-expression negatively influences cancer growth and invasion. On the other hand, the absence of Notch1 signaling in CAF leads to an increase in melanoma invasion [130].

CAFs also play an important role in oral cancer, where they frequently exhibit an expression of α-smooth muscle actin (α-SMA). It was shown that an upregulation of integrin-α6 in combination with α-SMA, correlates with a poor prognosis for patients [131,132] and that CAFs expressing α-SMA were shown to be involved in lymph node metastasis in oral squamous cell carcinoma [133,134]. As previously mentioned, the secretion of chemokines, cytokines, and growth factors by CAFs promotes the degradation of the extracellular matrix through release of matrix metalloproteinase (MPP), which is increased in HNSCC (Figure 3). Additionally, it was shown that the secretion of hepatocyte growth factor (HGF) by CAFs leads to an increase in VEGF and IL-8, hence an increase in angiogenesis. It also drives cell proliferation, migration, and invasion in HNSCC [135,136].

Summarizing, these factors emphasize the importance of the tumor microenvironment and especially the role of CAFs in oral cancer progression.

In regard to tumor-suppressing roles, it could be shown that blockage of NF-kB, as well as Ras, leads to invasive epidermal neoplasia mediated by the activity of tumor necrosis factor/c-Jun N-terminal kinase (TNF/JNK) [137]. As NF-kB activation is triggered by Notch signaling, it raises the question if Notch functions as a tumor suppressor via NF-kB activation in keratinocytes.

In a model of Notch1-deficient mice, basal cell carcinoma-like tumors arise spontaneously and were associated with the activation of sonic hedgehog (Shh) signaling. Additionally, an increase in β-catenin expression in the epidermis could be observed and reverted by active Notch1 [138]. These findings are supported by reports of reduced expression of Notch1, 2, and Jagged1 in basal cell carcinomas of human samples [139]. In OSCC, the expression of the Notch1 gene is variably reduced, as well as downstream target genes such as Hey1 [140]. In HNSCC cell lines, the overexpression of the active portion of Notch1 blocks cell cycle progression and produces a growth arrest. Similarly, in an in vivo model for HNSCC, activation of Notch1 resulted in reduced tumorigenicity [141].

In conclusion, the functional consequences of Notch activation or inhibition strongly depend on the tissue involved, the stage of cancer progression, and the single microenvironment features embedding the tumor. Additionally, the variety of levels in which the Notch pathway activation can be fine-tuned increases the complexity of the readout. A more detailed characterization of the conditions in which Notch act as oncogene or tumor-suppressor is essential to develop efficient future approaches for personalized medicine.

### 1.9. Crosstalk between Notch and Other Major Pathways

The complex alterations described in tumor development are often the result of multipathway misregulation. Crosstalk of Notch and other molecular pathway is of central importance in aberrant proliferation, apoptosis, and invasive phenotype.

The protein p53 is a pivotal tumor suppressor. It can be activated by diverse stress signals to ultimately modulate cellular responses such as transient cell cycle arrest, senescence, and apoptosis [142]. Crosstalk between p53 and the Notch pathway occurs at multiple levels. In an animal model for T-ALL, p53 levels are reduced due to Notch1 activation. Notch1 activation leads to an increased level of the E3 ubiquitin ligase mouse double minute homolog 2 (MDM2), which targets p53 for subsequent degradation [143] (Figure 4). Furthermore, in vitro experiments using breast cancer as well as keratinocyte cell lines show that activation of Notch causes an increased activity of phosphoinositide 3-kinase v-Akt murine thymoma viral oncogene (PI3K-Akt) pathway (Figure 4). This activation results in increased cell survival in response to MDM2 activity and subsequent reduction of p53 protein [144,145]. Aside of indirect up or downregulation of p53 by Notch activity, a direct interaction of Notch and p53 has been proposed, leading to p53 inhibition of phosphorylation and its DNA binding capability [146]. On the other hand, p53 activity increases upon induced expression of the Notch downstream effectors HEY1 and HES1, which leads to reduced MDM2 expression [147]. Noteworthy, an increase in p53 expression has been correlated with Notch1-dependent apoptosis and growth arrest in different tumor cell types, such as leukemia, hepatocellular cancer cells, and oral tongue cancer cells [148,149,150]. Aberration in the EGFR-PI3K-AKT pathways is a hallmark for oral cancer, where the cytoplasmatic phosphorylated form of AKT is expressed in more than 64% of cases [151,152,153] (Figure 4). In parallel, levels of expression of the receptor for EGF indicate a direct correspondence with tumor size and stage. Blockage of AKT and PI3K phosphorylation induces cell cycle arrest and apoptosis in OSCC cells [154]. During normal and malignant thymocytes development, Notch and PI3K pathway crosstalk via Hes1 and the PI3K negative regulator phosphatase and tensin homolog (PTEN). The downstream target Hes1 might directly downregulate expression of PTEN, increasing PI3K activity [155] (Figure 4). In a screening for Notch1 inactivating mutation, the oncogenic phenotype was associated with activation of the EGF-PI3K/AKT pathway and resulted in increased cell proliferation, EMT, and invasion in OSCC cell lines [156].

Like the Notch signaling pathway, Wnt signaling is implicated in organ development and regulation of stemness. Crosstalk between Wnt and Notch signaling has been reported in different types of cancer [157,158]. In these settings, β-catenin/ T-cell factor/lymphoid enhancer factor (TCF) causes an activation of the Notch signaling pathway and leads to expression of the downstream oncogenes Myc proto-oncogene (Myc) [158], Hes1, cluster of differentiation 44 (CD44), nicotidamide adenine dinucleotide phosphate oxidase 1 (Nox1), SOX9, ephrin type-B receptor 3 (EphHB3), and Kruppel like factor 5 (KLF5) [157]. In OSCC, activation of the Wnt pathway can occur in absence of β-catenin and colon-cancer specific mutations, suggesting that altered epigenetic changes might compensate for the canonical activation of the pathway [159] (Figure 4).

During embryonic development, the Notch pathway regulates fate determination and cell number in cooperation with the Hedgehog (HH) pathway. Mutations in HH-pathways have been found in basal cell carcinoma, medulloblastoma, and rhabdomyosarcoma, and normally correlate with an hyperactivation of the pathway. Recent studies on protein expression showed that SHH signaling components are highly expressed in OSCC compared to normal tissue, with a restricted positive pattern in epithelial cells [160,161,162,163].

SHH has been shown to act on the target cells to increase the transcription of several genes, including members of the Notch pathway. Additionally, the Notch pathway has been involved in the regulation of intracellular localization of SHH, suggesting a critical bidirectional control of the two pathways [164].

### 1.10. Current Animal Models for Oral Cancer

In the recent years, due to the development of new methods and techniques, several animal models have been generated to study the initiation, formation and progression of HNSCC in vivo.

#### 1.10.1. Chemically Induced Mouse Models of Oral Cancer

The chemical 4-nitroquinoline 1-oxide (4NQO) is a carcinogenic and mutagenic quinoline. This chemical is used to generate an oral cancer model in mice and rats. 4-NQO is administered either locally on the tongue or adding 4NQO to the drinking water [165,166,167]. Long-term administration of 4-NQO leads to a progression of carcinogenesis and includes hyperplasia, dysplasia, preneoplastic as well as neoplastic lesions. The advantage of this model is to efficiently reproduce many characteristic features of the human oral cancer development and progression. Molecular analyses of pathways and testing novel therapeutic approaches can therefore be assessed at different stages of the disease progression [3,4,167,168]. This model allows studies on tumor microenvironment as well as vascularization, preserving the complex interaction of cell-to-cell communication and cell-to-extracellular environment supporting tumor growth [168].

#### 1.10.2. Transgenic Murine Models

The first developed genetic mouse model to study the development of oral-esophageal squamous epithelium was generated exploiting the promoter of Epstein-Barr virus ED-L2 (L2). As the p53 gene is mutated in 70% of HNSCC [169], this mouse was further crossed with p53^+/−^ and p53^−/−^ mice, resulting in mice that develop invasive oral-esophageal squamous cell carcinoma. The final model (L2D1/p53) enabled to study the roles of EGFR, p53, and CDK4 genes and their influence in the formation of HNSCC [170]. The Tgfbr1/Pten 2cKO mice have an inducible control over the expression of Tgfbr1/Pten genes [171,172]. Phosphatase and tension homolog (PTEN) is a tumor suppressor gene and thought to play a key role in HNSCC. TGF-β signaling is known to have tumor-suppressing as well as tumor-promoting roles in various cancers. Upon induced-ablation of the two genes, mice develop hyperplasia in the oral epithelium [172]. The loss of Tgfbr1 and Pten was shown to lead to cancer-related inflammation as well as cancer stem cell expansion in the basal epithelial layer of this model [172]. Finally, a mutated form of Kirsten rat sarcoma viral oncogene homolog (K-Ras) was expressed under the control of cytokeratin 5 and cytokeratin 14 (cK5 and cK14). The expression of the two cytokeratin is confined to the stratified epithelia of the oral cavity, allowing a conditional expression of the oncogene limited to the area of interest. Both mice models develop oral papilloma, with some tissue specificity. Overexpression of Kras^G12D^ under the control of cK5 affects the basal epithelium, while under the regulation of cK14, alterations are mainly found in the basal layer of oral mucosa and the tongue [173,174].

### 1.11. Therapeutic Strategies to Target Notch Signaling

As previously discussed, the cancer stem cells have the capability of self-renewal and amplify the subset of undifferentiated progenitor cells. Notch is one of the key players in the maintenance of cancer stem cells and it is involved in the regulation of motility and structural changes supporting metastasis. Additionally, Notch plays a pivotal role in angiogenesis, critical for maintenance and progression of tumorigenesis. Therefore, targeting Notch to eliminate cancer stem cells has become one of the main pharmacological strategies to fight cancer [175]. On the other hand, Notch is essential for the maintenance of homeostasis in a variety of tissues, and the development of agents with minimized off-target side-effects requires a deeper understanding of the specific role of Notch in cancer. Two main strategies to target Notch signaling exist: (i) interference with Notch ligand–receptor interaction, such as the use of monoclonal antibodies targeting specific regions for ligand–receptor binding or (ii) preventing Notch receptor cleavages, inhibiting down-stream gene transcription (such as GSI DAPT). Promising antitumor activity observations were made in experiments where GSI DAPT and monoclonal antibodies (mAbs), were administered as a combined treatment. The antitumorigenic effects could be detected in early stages of, e.g., lung cancer, sarcoma, colorectal cancer, thyroid cancer [176]. Inhibiting Notch1 in an HNSCC xenograft mouse model resulted in reduced cancer stem cell renewal. Furthermore, the combinatory use of chemotherapeutic drugs and Notch inhibitor caused a reduction in the cancer stem cell population in vitro and in vivo. Another prominent target to inhibit Notch signaling in cancer is the ligand DLL-4. Chemotherapy or radiotherapy in combination with mAb against DLL-4 promoted tumor necrosis. This approach also reduced the frequency of tumor initiating cells, impaired tumor angiogenesis, and delayed tumor relapse [78]. However, using mAbs or GSI DAPTs causes severe side effects and requires periodic administration with lag phases for patient recovery.

Additionally to chemotherapeutic agents, natural compounds and phytochemicals possess useful anticancer properties that reduce cells proliferation, cancer stem cell renewal, and induce apoptosis [177,178,179]. Alternative strategies of chemoprevention have been gaining increasing interest during the last years due to their low toxicity and safeness over long term exposure. Specifically, several natural compounds have been used to contrast various forms of carcinomas. *Xanthohumol* (*XN*), derived from the humulus lupulus plant, and *paeoniflorin* (*PF*), from the Chinese peony, decrease Notch signaling activation in a mouse model of breast cancer inducing apoptosis of tumorigenic cells and suppressing proliferation [180,181]. Similarly, *diallyl trisulfide* (*DATS*), found in garlic, inhibits the expression of ADAM10 and ADAM17 responsible for Notch signaling pathway activation in transformed breast epithelial cells [182].

In small cell lung cancer (NSCLC) the flavone *baicalein* was found to reduce proliferation in vitro as consequence of Notch1 and Hes1 downregulation of expression [183]. In both colon cancer and melanoma, *honokiol* and *withaferin A* reduce Notch1, Jagged1, and Hes1 expression, inhibiting carcinogenesis [184,185,186]. *Curcumin* is one of the most effective chemopreventive agents in oral cancer. Curcumin is a polyphenolic derivate of turmeric from curcuma longa, and its anticancer activity has been associated with the interference of the Notch-NF-κB-cyclinD1 axis. Curcumin downregulates Notch1, which in turn inhibits NF-κB activation, resulting in a blockage of the downstream targets VEGF and CyclinD1, involved respectively in tumor-angiogenesis and proliferation [187]. A similar mechanism has been found in esophageal cancer cells and, more generally, might affect the formation of cancer stem cells [177,188].

Summarizing, the inhibition of Notch signaling in malignant tumor enables a broad set of approaches for future therapeutic strategies. Refining the spectrum of pharmacological targets is the key factor to efficiently direct the treatment to the tumorigenic core.

## 2. Concluding Remarks

The Notch pathway is a well conserved molecular pathway essential for tissue homeostasis. HNSCC is one of the most common solid tumors, in which the Notch pathway is altered; with severe consequences for cellular proliferation and migration. Despite the urgent need for treatments contrasting HNSCC, we are still lacking basic knowledge regarding the initiation and evolution of the disease. As summarized in this review, the Notch pathway is involved in all major tumorigenic events occurring during HNSCC development, including aberrant angiogenesis, regulation of stem cell renewal, proliferation, and invasion. It therefore represents a central molecular element at the basis of tumor formation and provides a promising therapeutic target for future HNSCC therapies.

## Abbreviations

ADAM	A Disintegrin And Metalloproteinase
APC	Adenomatous polyposis coli
CAF	Cancer-associated fibroblast
CBF1	C promoter-binding factor 1
CD44	Cluster of differentiation 44
cK	Cytokeratin
CSC	Cancer stem cell
CUP	Carcinoma of unknown primary
CXCR4	CXC chemokine receptor 4
DC	Dendritic cells
DLL	Delta-like
EGF(R)	Epidermal growth factor (receptor)
EMTEphHB3	Epithelial-to-mesenchymal transitionEphrin type-B receptor 3
ERK	Extracellular regulated kinase
GSI DAPT	Gamma-secretase inhibitors N-[N-(3,5-difluorophenacetyl)-l-alanyl]-S-phenylglycine t-butyl ester
Hes	Hairy and enhancer of split
Hey	Hairy/enhancer-of-split related with YRPW motif
HGF	Hepatocyte growth factor
HH	Hedgehog
Hif1a	Hypoxia-inducible factor 1 alpha subunit
HNSCC	Head and neck squamous cell carcinoma
Ikk2	Inhibitor of nuclear factor kappa-B kinase subunit kinase β
IL	Interleukin
ILC	Innate lymphoid cell
JNK	c-Jun N-terminal kinase
KLF5	Kruppel like factor 5
K-Ras	Kirsten rat sarcoma viral oncogene homolog
Lfng	Lunatic Fringe
LNR	Cysteine-rich Lin-12/Notch Repeats
mAb	Monoclonal antibody
MAPK	Mitogen-activated protein kinase
MDM2	Mouse double minute homolog 2
MMP	Matrix metalloproteinase
Mnfg	Maniac Fringe
Myc	Myc proto-oncogene
NCR	Natural cytotoxicity receptor
NECD	Notch extracellular domain
NEXT	Notch extracellular truncation
NF-κB	Nuclear factor kappa-light-chain-enhancer of activated B cells
NICD	Notch intracellular domain
NK	Natural killer cells
NLS	Nuclear localizing sequence
Nox1	Nicotidamide adenine dinucleotide phosphate oxidase 1
OCT4	Octamer-binding transcription factor 4
OSCC	Oral Squamous Cell Carcinoma
PI3K-AKT	Phosphoinositide 3-kinase v-Akt murine thymoma viral oncogene
Pparg	Peroxisome proliferator-activated receptor gamma
PTEN	Phosphatase and tensin homolog
RBPj	Recombining binding protein suppressor of hairless
Rfng	Radical Fringe
Shh	Sonic hedgehog
STAT3	Signal transducer and activator of transcription 3
SOX2	Sex determining region Y-box 2
TAM	Tumor associated macrophages
TCF	T-cell factor/lymphoid enhancer factor
T-ALL	T cell acute lymphoblastic leukemia
TCF	T-cell factor
TGF-β	Transforming growth factor-β
Tgfbr1	Transforming growth factor-β receptor 1
TNF-α	Tumor necrosis factor alpha
VEGF(R)	Vascular endothelial growth factor (receptor)
WHO	World Health Organization
Wnt	Wingless/Integrated
α-SMA	α-smooth muscle actin
4NQO	4-nitroquinoline 1-oxide

## References

[1] Sanderson R.J., Ironside J.A.D. (2002). Squamous cell carcinomas of the head and neck. BMJ.

[2] Vargas H., Pitman K.T., Johnson J.T., Galati L.T. (2000). More aggressive behavior of squamous cell carcinoma of the anterior tongue in young women. Laryngoscope.

[3] Gilroy J.S., Morris C.G., Amdur R.J., Mendenhall W.M. (2005). Impact of young age on prognosis for head and neck cancer: A matched-pair analysis. Head Neck.

[4] Goldenberg D., Brooksby C., Hollenbeak C.S. (2009). Age as a determinant of outcomes for patients with oral cancer. Oral Oncol..

[5] Ascani G., Balercia P., Messi M., Lupi L., Goteri G., Filosa A., Stramazzotti D., Pieramici T., Rubini C. (2005). Angiogenesis in oral squamous cell carcinoma. Acta Otorhinol. Ital. Organo Uff. Della Soc. Ital. Otorinolaringol. E Chir. Cerv.-Facc..

[6] Warnakulasuriya S. (2009). Global epidemiology of oral and oropharyngeal cancer. Oral Oncol..

[7] Leethanakul C., Patel V., Gillespie J., Pallente M., Ensley J.F., Koontongkaew S., Liotta L.A., Emmert-Buck M., Gutkind J.S. (2000). Distinct pattern of expression of differentiation and growth-related genes in squamous cell carcinomas of the head and neck revealed by the use of laser capture microdissection and cDNA arrays. Oncogene.

[8] Baik F.M., Hansen S., Knoblaugh S.E., Sahetya D., Mitchell R.M., Xu C., Olson J.M., Parrish-Novak J., Méndez E. (2016). Fluorescence Identification of Head and Neck Squamous Cell Carcinoma and High-Risk Oral Dysplasia With BLZ-100, a Chlorotoxin-Indocyanine Green Conjugate. JAMA Otolaryngol. Head Neck Surg..

[9] Gillespie M.B., Albergotti W.G., Eisele D.W. (2012). Recurrent salivary gland cancer. Curr. Treat. Options Oncol..

[10] Patel V.N., Hoffman M.P. (2014). Salivary gland development: A template for regeneration. Semin. Cell Dev. Biol..

[11] Emmerson E., Knox S.M. (2018). Salivary gland stem cells: A review of development, regeneration and cancer. Genesis.

[12] Konings A.W.T., Coppes R.P., Vissink A. (2005). On the mechanism of salivary gland radiosensitivity. Int. J. Radiat. Oncol. Biol. Phys..

[13] Konings A.W.T., Faber H., Cotteleer F., Vissink A., Coppes R.P. (2006). Secondary radiation damage as the main cause for unexpected volume effects: A histopathologic study of the parotid gland. Int. J. Radiat. Oncol. Biol. Phys..

[14] Cooper J.S., Fu K., Marks J., Silverman S. (1995). Late effects of radiation therapy in the head and neck region. Int. J. Radiat. Oncol. Biol. Phys..

[15] Saman D.M. (2012). A review of the epidemiology of oral and pharyngeal carcinoma: Update. Head Neck Oncol..

[16] Agrawal N., Frederick M.J., Pickering C.R., Bettegowda C., Chang K., Li R.J., Fakhry C., Xie T.X., Zhang J., Wang J., Zhang N. (2011). Exome sequencing of head and neck squamous cell carcinoma reveals inactivating mutations in NOTCH1. Science.

[17] Huber M.A., Tantiwongkosi B. (2014). Oral and oropharyngeal cancer. Med. Clin. North Am..

[18] Gillison M.L., Broutian T., Pickard R.K., Tong Z.Y., Xiao W., Kahle L., Graubard B.I., Chaturvedi A.K. (2012). Prevalence of oral HPV infection in the United States, 2009–2010. JAMA.

[19] Bernier J., Cooper J.S., Pajak T.F., van Glabbeke M., Bourhis J., Forastiere A., Ozsahin E.M., Jacobs J.R., Jassem J., Ang K.K., Lefèbvre J.L. (2005). Defining risk levels in locally advanced head and neck cancers: A comparative analysis of concurrent postoperative radiation plus chemotherapy trials of the EORTC (#22931) and RTOG (# 9501). Head Neck.

[20] Sommers L.W., Steenbakkers R.J.H.M., Bijl H.P., Vemer-van den Hoek J.G.M., Roodenburg J.L.N., Oosting S.F., Halmos G.B., de Rooij S.E., Langendijk J.A. (2017). Survival Patterns in Elderly Head and Neck Squamous Cell Carcinoma Patients Treated with Definitive Radiation Therapy. Int. J. Radiat. Oncol. Biol. Phys..

[21] Artavanis-Tsakonas S., Rand M.D., Lake R.J. (1999). Notch signaling: Cell fate control and signal integration in development. Science.

[22] Meloty-Kapella L., Shergill B., Kuon J., Botvinick E., Weinmaster G. (2012). Notch ligand endocytosis generates mechanical pulling force dependent on dynamin, epsins, and actin. Dev. Cell.

[23] Ahimou F., Mok L.-P., Bardot B., Wesley C. (2004). The adhesion force of Notch with Delta and the rate of Notch signaling. J. Cell Biol..

[24] Huenniger K., Krämer A., Soom M., Chang I., Köhler M., Depping R., Kehlenbach R.H., Kaether C. (2010). Notch1 signaling is mediated by importins alpha 3, 4, and 7. Cell. Mol. Life Sci. CMLS.

[25] Liu Z., Teng L., Bailey S.K., Frost A.R., Bland K.I., LoBuglio A.F., Ruppert J.M., Lobo-Ruppert S.M. (2009). Epithelial transformation by KLF4 requires Notch1 but not canonical Notch1 signaling. Cancer Biol. Ther..

[26] Raafat A., Lawson S., Bargo S., Klauzinska M., Strizzi L., Goldhar A.S., Buono K., Salomon D., Vonderhaar B.K., Callahan R. (2009). Rbpj conditional knockout reveals distinct functions of Notch4/Int3 in mammary gland development and tumorigenesis. Oncogene.

[27] Lahmar M., Catelain C., Poirault S., Dorsch M., Villeval J.L., Vainchenker W., Albagli O., Lauret E. (2008). Distinct effects of the soluble versus membrane-bound forms of the notch ligand delta-4 on human CD34+CD38low cell expansion and differentiation. Stem Cells Dayt. Ohio.

[28] Liao W.R., Hsieh R.H., Hsu K.W., Wu M.Z., Tseng M.J., Mai R.T., Wu Lee Y.H., Yeh T.S. (2007). The CBF1-independent Notch1 signal pathway activates human c-myc expression partially via transcription factor YY1. Carcinogenesis.

[29] Ikawa T., Kawamoto H., Goldrath A.W., Murre C. (2006). E proteins and Notch signaling cooperate to promote T cell lineage specification and commitment. J. Exp. Med..

[30] Stockhausen M.-T., Sjölund J., Axelson H. (2005). Regulation of the Notch target gene Hes-1 by TGFalpha induced Ras/MAPK signaling in human neuroblastoma cells. Exp. Cell Res..

[31] Nichols J.T., Miyamoto A., Weinmaster G. (2007). Notch signaling—Constantly on the move. Traffic.

[32] Panin V.M., Papayannopoulos V., Wilson R., Irvine K.D. (1997). Fringe modulates Notch-ligand interactions. Nature.

[33] Cohen B., Bashirullah A., Dagnino L., Campbell C., Fisher W.W., Leow C.C., Whiting E., Ryan D., Zinyk D., Boulianne G. (1997). Fringe boundaries coincide with Notch-dependent patterning centres in mammals and alter Notch-dependent development in Drosophila. Nat. Genet..

[34] Xu K., Usary J., Kousis P.C., Prat A., Wang D.Y., Adams J.R., Wang W., Loch A.J., Deng T., Zhao W. (2012). Lunatic fringe deficiency cooperates with the Met/Caveolin gene amplicon to induce basal-like breast cancer. Cancer Cell.

[35] Collins L.M., Dawes C. (1987). The surface area of the adult human mouth and thickness of the salivary film covering the teeth and oral mucosa. J. Dent. Res..

[36] Zhang T.H., Liu H.C., Zhu L.J., Chu M., Liang Y.J., Liang L.Z., Liao G.Q. (2011). Activation of Notch signaling in human tongue carcinoma. J. Oral Pathol. Med..

[37] Mandasari M., Sawangarun W., Katsube K., Kayamori K., Yamaguchi A., Sakamoto K. (2016). A facile one-step strategy for the generation of conditional knockout mice to explore the role of Notch1 in oroesophageal tumorigenesis. Biochem. Biophys. Res. Commun..

[38] Zhao Z.L., Zhang L., Huang C.F., Ma S.R., Bu L.L., Liu J.F., Yu G.T., Liu B., Gutkind J.S., Kulkarni A.B., Zhang W.F., Sun Z.J. (2016). NOTCH1 inhibition enhances the efficacy of conventional chemotherapeutic agents by targeting head neck cancer stem cell. Sci. Rep..

[39] Uhlén M., Fagerberg L., Hallström B.M., Lindskog C., Oksvold P., Mardinoglu A., Sivertsson Å., Kampf C., Sjöstedt E., Asplund A. (2015). Proteomics. Tissue-based map of the human proteome. Science.

[40] Uhlen M., Oksvold P., Fagerberg L., Lundberg E., Jonasson K., Forsberg M., Zwahlen M., Kampf C., Wester K., Hober S. (2010). Towards a knowledge-based Human Protein Atlas. Nat. Biotechnol..

[41] Zhang T.H., Liu H.C., Liang Y.J., Liang L.Z., Zheng G.S., Huang H.Z., Wu J.N., Liao G.Q. (2013). Suppression of tongue squamous cell carcinoma growth by inhibition of Jagged1 in vitro and in vivo. J. Oral Pathol. Med..

[42] Dang H., Lin A.L., Zhang B., Zhang H.-M., Katz M.S., Yeh C.-K. (2009). Role for Notch signaling in salivary acinar cell growth and differentiation. Dev. Dyn. Off. Publ. Am. Assoc. Anat..

[43] Jiang R., Lan Y., Chapman H.D., Shawber C., Norton C.R., Serreze D.V., Weinmaster G., Gridley T. (1998). Defects in limb, craniofacial, and thymic development in Jagged2 mutant mice. Genes Dev..

[44] Casey L.M., Lan Y., Cho E.-S., Maltby K.M., Gridley T., Jiang R. (2006). Jag2-Notch1 signaling regulates oral epithelial differentiation and palate development. Dev. Dyn. Off. Publ. Am. Assoc. Anat..

[45] Kamath B.M., Bauer R.C., Loomes K.M., Chao G., Gerfen J., Hutchinson A., Hardikar W., Hirschfield G., Jara P., Krantz I.D. (2012). NOTCH2 mutations in Alagille syndrome. J. Med. Genet..

[46] Mitsiadis T.A., Graf D., Luder H., Gridley T., Bluteau G. (2010). BMPs and FGFs target Notch signalling via jagged 2 to regulate tooth morphogenesis and cytodifferentiation. Dev. Camb. Engl..

[47] Mitsiadis T.A., Henrique D., Thesleff I., Lendahl U. (1997). Mouse Serrate-1 (Jagged-1): Expression in the developing tooth is regulated by epithelial-mesenchymal interactions and fibroblast growth factor-4. Dev. Camb. Engl..

[48] Mitsiadis T.A., de Bari C., About I. (2008). Apoptosis in developmental and repair-related human tooth remodeling: A view from the inside. Exp. Cell Res..

[49] Mitsiadis T.A., Graf D. (2009). Cell fate determination during tooth development and regeneration. Birth Defects Res. Part C Embryo Today Rev..

[50] Mitsiadis T.A., Lardelli M., Lendahl U., Thesleff I. (1995). Expression of Notch 1, 2 and 3 is regulated by epithelial-mesenchymal interactions and retinoic acid in the developing mouse tooth and associated with determination of ameloblast cell fate. J. Cell Biol..

[51] Hill-Felberg S., Wu H.H., Toms S.A., Dehdashti A.R. (2015). Notch receptor expression in human brain arteriovenous malformations. J. Cell. Mol. Med..

[52] Conboy I.M., Rando T.A. (2005). Aging, stem cells and tissue regeneration: Lessons from muscle. Cell Cycle.

[53] Church J.E., Trieu J., Chee A., Naim T., Gehrig S.M., Lamon S., Angelini C., Russell A.P., Lynch G.S. (2014). Alterations in Notch signalling in skeletal muscles from mdx and dko dystrophic mice and patients with Duchenne muscular dystrophy. Exp. Physiol..

[54] Van den Engel-Hoek L., Erasmus C.E., Hendriks J.C., Geurts A.C., Klein W.M., Pillen S., Sie L.T., de Swart B.J., de Groot I.J. (2013). Oral muscles are progressively affected in Duchenne muscular dystrophy: Implications for dysphagia treatment. J. Neurol..

[55] Vieira N.M., Elvers I., Alexander M.S., Moreira Y.B., Eran A., Gomes J.P., Marshall J.L., Karlsson E.K., Verjovski-Almeida S., Lindblad-Toh K. (2015). Jagged 1 Rescues the Duchenne Muscular Dystrophy Phenotype. Cell.

[56] Marelli F., Persani L. (2018). Role of Jagged1-Notch pathway in thyroid development. J. Endocrinol. Investig..

[57] Kumar L.K.S., Kurien N.M., Jacob M.M., Menon P.V., Khalam S.A. (2015). Lingual thyroid. Ann. Maxillofac. Surg..

[58] Demehri S., Turkoz A., Kopan R. (2009). Epidermal Notch1 loss promotes skin tumorigenesis by impacting the stromal microenvironment. Cancer Cell.

[59] Liu Y., Liu C.-X., Wu Z.-T., Ge L., Zhou H.-M. (2013). Mining proteins associated with oral squamous cell carcinoma in complex networks. Asian Pac. J. Cancer Prev. APJCP.

[60] Sakamoto K. (2016). Notch signaling in oral squamous neoplasia. Pathol. Int..

[61] Sun W., Gaykalova D.A., Ochs M.F., Mambo E., Arnaoutakis D., Liu Y., Loyo M., Agrawal N., Howard J., Li R. (2014). Activation of the NOTCH pathway in head and neck cancer. Cancer Res..

[62] Frise E., Knoblich J.A., Younger-Shepherd S., Jan L.Y., Jan Y.N. (1996). The Drosophila Numb protein inhibits signaling of the Notch receptor during cell-cell interaction in sensory organ lineage. Proc. Natl. Acad. Sci. USA.

[63] Guo M., Jan L.Y., Jan Y.N. (1996). Control of daughter cell fates during asymmetric division: Interaction of Numb and Notch. Neuron.

[64] Berdnik D., Török T., González-Gaitán M., Knoblich J.A. (2002). The endocytic protein alpha-Adaptin is required for numb-mediated asymmetric cell division in Drosophila. Dev. Cell.

[65] Hong J., Liu Z., Zhu H., Zhang X., Liang Y., Yao S., Wang F., Xie X., Zhang B., Tan T. (2014). The tumor suppressive role of NUMB isoform 1 in esophageal squamous cell carcinoma. Oncotarget.

[66] Hung P.S., Liu C.J., Chou C.S., Kao S.Y., Yang C.C., Chang K.W., Chiu T.H., Lin S.C. (2013). miR-146a enhances the oncogenicity of oral carcinoma by concomitant targeting of the IRAK1, TRAF6 and NUMB genes. PLoS ONE.

[67] Hijioka H., Setoguchi T., Miyawaki A., Gao H., Ishida T., Komiya S., Nakamura N. (2010). Upregulation of Notch pathway molecules in oral squamous cell carcinoma. Int. J. Oncol..

[68] Simabuco F.M., Kawahara R., Yokoo S., Granato D.C., Miguel L., Agostini M., Aragão A.Z., Domingues R.R., Flores I.L., Macedo C.C. (2014). ADAM17 mediates OSCC development in an orthotopic murine model. Mol. Cancer.

[69] Hellström M., Phng L.K., Hofmann J.J., Wallgard E., Coultas L., Lindblom P., Alva J., Nilsson A.K., Karlsson L., Gaiano N. (2007). Dll4 signalling through Notch1 regulates formation of tip cells during angiogenesis. Nature.

[70] Suchting S., Freitas C., le Noble F., Benedito R., Bréant C., Duarte A., Eichmann A. (2007). The Notch ligand Delta-like 4 negatively regulates endothelial tip cell formation and vessel branching. Proc. Natl. Acad. Sci. USA.

[71] Leslie J.D., Ariza-McNaughton L., Bermange A.L., McAdow R., Johnson S.L., Lewis J. (2007). Endothelial signalling by the Notch ligand Delta-like 4 restricts angiogenesis. Dev. Camb. Engl..

[72] Siekmann A.F., Lawson N.D. (2007). Notch signalling limits angiogenic cell behaviour in developing zebrafish arteries. Nature.

[73] Williams C.K., Li J.-L., Murga M., Harris A.L., Tosato G. (2006). Up-regulation of the Notch ligand Delta-like 4 inhibits VEGF-induced endothelial cell function. Blood.

[74] Benedito R., Roca C., Sörensen I., Adams S., Gossler A., Fruttiger M., Adams R.H. (2009). The notch ligands Dll4 and Jagged1 have opposing effects on angiogenesis. Cell.

[75] Mazzieri R., Pucci F., Moi D., Zonari E., Ranghetti A., Berti A., Politi L.S., Gentner B., Brown J.L., Naldini L. (2011). Targeting the ANG2/TIE2 axis inhibits tumor growth and metastasis by impairing angiogenesis and disabling rebounds of proangiogenic myeloid cells. Cancer Cell.

[76] Outtz H.H., Wu J.K., Wang X., Kitajewski J. (2010). Notch1 deficiency results in decreased inflammation during wound healing and regulates vascular endothelial growth factor receptor-1 and inflammatory cytokine expression in macrophages. J. Immunol..

[77] Patel N.S., Li J.-L., Generali D., Poulsom R., Cranston D.W., Harris A.L. (2005). Up-regulation of delta-like 4 ligand in human tumor vasculature and the role of basal expression in endothelial cell function. Cancer Res..

[78] Noguera-Troise I., Daly C., Papadopoulos N.J., Coetzee S., Boland P., Gale N.W., Lin H.C., Yancopoulos G.D., Thurston G. (2006). Blockade of Dll4 inhibits tumour growth by promoting non-productive angiogenesis. Nature.

[79] Zaghloul N., Hernandez S.L., Bae J.O., Huang J., Fisher J.C., Lee A., Kadenhe-Chiweshe A., Kandel J.J., Yamashiro D.J. (2009). Vascular endothelial growth factor blockade rapidly elicits alternative proangiogenic pathways in neuroblastoma. Int. J. Oncol..

[80] Hasina R., Lingen M.W. (2001). Angiogenesis in oral cancer. J. Dent. Educ..

[81] Zeng Q., Li S., Chepeha D.B., Giordano T.J., Li J., Zhang H., Polverini P.J., Nor J., Kitajewski J., Wang C.Y. (2005). Crosstalk between tumor and endothelial cells promotes tumor angiogenesis by MAPK activation of Notch signaling. Cancer Cell.

[82] Kayamori K., Katsube K., Sakamoto K., Ohyama Y., Hirai H., Yukimori A., Ohata Y., Akashi T., Saitoh M., Harada K. (2016). NOTCH3 Is Induced in Cancer-Associated Fibroblasts and Promotes Angiogenesis in Oral Squamous Cell Carcinoma. PLoS ONE.

[83] Wang W.M., Zhao Z.L., Ma S.R., Yu G.T., Liu B., Zhang L., Zhang W.F., Kulkarni A.B., Sun Z.J., Zhao Y.F. (2015). Epidermal growth factor receptor inhibition reduces angiogenesis via hypoxia-inducible factor-1α and Notch1 in head neck squamous cell carcinoma. PLoS ONE.

[84] Chan C.J., Heisenberg C.-P., Hiiragi T. (2017). Coordination of Morphogenesis and Cell-Fate Specification in Development. Curr. Biol..

[85] Voulgari A., Pintzas A. (2009). Epithelial-mesenchymal transition in cancer metastasis: Mechanisms, markers and strategies to overcome drug resistance in the clinic. Biochim. Biophys. Acta.

[86] Fragiadaki M., Mason R.M. (2011). Epithelial-mesenchymal transition in renal fibrosis-evidence for and against. Int. J. Exp. Pathol..

[87] Thiery J.P. (2002). Epithelial-mesenchymal transitions in tumour progression. Nat. Rev. Cancer.

[88] Tse J.C., Kalluri R. (2007). Mechanisms of metastasis: Epithelial-to-mesenchymal transition and contribution of tumor microenvironment. J. Cell. Biochem..

[89] Gravdal K., Halvorsen O.J., Haukaas S.A., Akslen L.A. (2007). A switch from E-cadherin to N-cadherin expression indicates epithelial to mesenchymal transition and is of strong and independent importance for the progress of prostate cancer. Clin. Cancer Res. Off. J. Am. Assoc. Cancer Res..

[90] Yang Z., Zhang X., Gang H., Li X., Li Z., Wang T., Han J., Luo T., Wen F., Wu X. (2007). Up-regulation of gastric cancer cell invasion by Twist is accompanied by N-cadherin and fibronectin expression. Biochem. Biophys. Res. Commun..

[91] Saad S., Stanners S.R., Yong R., Tang O., Pollock C.A. (2010). Notch mediated epithelial to mesenchymal transformation is associated with increased expression of the Snail transcription factor. Int. J. Biochem. Cell Biol..

[92] Stoyianni A., Goussia A., Pentheroudakis G., Siozopoulou V., Ioachim E., Krikelis D., Golfinopoulos V., Cervantes A., Bobos M., Fotsis T. (2012). Immunohistochemical study of the epithelial-mesenchymal transition phenotype in cancer of unknown primary: Incidence, correlations and prognostic utility. AntiCancer Res..

[93] Xie M., Zhang L., He C.S., Xu F., Liu J.L., Hu Z.H., Zhao L.P., Tian Y. (2012). Activation of Notch-1 enhances epithelial-mesenchymal transition in gefitinib-acquired resistant lung cancer cells. J. Cell. Biochem..

[94] Ishida T., Hijioka H., Kume K., Miyawaki A., Nakamura N. (2013). Notch signaling induces EMT in OSCC cell lines in a hypoxic environment. Oncol. Lett..

[95] Krisanaprakornkit S., Iamaroon A. (2012). Epithelial-mesenchymal transition in oral squamous cell carcinoma. ISRN Oncol..

[96] Bigas A., Porcheri C. (2018). Notch and Stem Cells. Adv. Exp. Med. Biol..

[97] Pannuti A., Foreman K., Rizzo P., Osipo C., Golde T., Osborne B., Miele L. (2010). Targeting Notch to target cancer stem cells. Clin. Cancer Res. Off. J. Am. Assoc. Cancer Res..

[98] Sansone P., Storci G., Giovannini C., Pandolfi S., Pianetti S., Taffurelli M., Santini D., Ceccarelli C., Chieco P., Bonafé M. (2007). p66Shc/Notch-3 interplay controls self-renewal and hypoxia survival in human stem/progenitor cells of the mammary gland expanded in vitro as mammospheres. Stem Cells Dayt. Ohio.

[99] Harrison H., Farnie G., Howell S.J., Rock R.E., Stylianou S., Brennan K.R., Bundred N.J., Clarke R.B. (2010). Regulation of breast cancer stem cell activity by signaling through the Notch4 receptor. Cancer Res..

[100] Fan X., Matsui W., Khaki L., Stearns D., Chun J., Li Y.M., Eberhart C.G. (2006). Notch pathway inhibition depletes stem-like cells and blocks engraftment in embryonal brain tumors. Cancer Res..

[101] Fan L., Liu Y., Ying H., Xue Y., Zhang Z., Wang P., Liu L., Zhang H. (2011). Increasing of blood-tumor barrier permeability through paracellular pathway by low-frequency ultrasound irradiation in vitro. J. Mol. Neurosci..

[102] Yao Z., Mishra L. (2009). Cancer stem cells and hepatocellular carcinoma. Cancer Biol. Ther..

[103] Wang M., Xue L., Cao Q., Lin Y., Ding Y., Yang P., Che L. (2009). Expression of Notch1, Jagged1 and beta-catenin and their clinicopathological significance in hepatocellular carcinoma. Neoplasma.

[104] Domingo-Domenech J., Vidal S.J., Rodriguez-Bravo V., Castillo-Martin M., Quinn S.A., Rodriguez-Barrueco R., Bonal D.M., Charytonowicz E., Gladoun N., de la Iglesia-Vicente J. (2012). Suppression of acquired docetaxel resistance in prostate cancer through depletion of notch- and hedgehog-dependent tumor-initiating cells. Cancer Cell.

[105] Crabtree J.S., Miele L. (2018). Breast Cancer Stem Cells. Biomedicines.

[106] Pandya K., Meeke K., Clementz A.G., Rogowski A., Roberts J., Miele L., Albain K.S., Osipo C. (2011). Targeting both Notch and ErbB-2 signalling pathways is required for prevention of ErbB-2-positive breast tumour recurrence. Br. J. Cancer.

[107] De Andrade N.P., Rodrigues S.D., Rodini C.O., Nunes F.D. (2017). Cancer stem Cell cytokeratins and epithelial to mesenchymal transition markers expression in oral squamous cell carcinoma derived from ortothopic xenoimplantation of CD44high cells. Pathol. Res. Pract..

[108] Chen J., Zhou J., Lu J., Xiong H., Shi X., Gong L. (2014). Significance of CD44 expression in head and neck cancer: A systemic review and meta-analysis. BMC Cancer.

[109] Zeineddine D., Hammoud A.A., Mortada M., Boeuf H. (2014). The Oct4 protein: More than a magic stemness marker. Am. J. Stem Cells.

[110] Huang C.-F., Xu X.-R., Wu T.-F., Sun Z.-J., Zhang W.-F. (2014). Correlation of ALDH1, CD44, OCT4 and SOX2 in tongue squamous cell carcinoma and their association with disease progression and prognosis. J. Oral Pathol. Med..

[111] Baillie R., Itinteang T., Yu H.H., Brasch H.D., Davis P.F., Tan S.T. (2016). Cancer stem cells in moderately differentiated oral tongue squamous cell carcinoma. J. Clin. Pathol..

[112] Zimmerer R.M., Ludwig N., Kampmann A., Bittermann G., Spalthoff S., Jungheim M., Gellrich N.C., Tavassol F. (2017). CD24+ tumor-initiating cells from oral squamous cell carcinoma induce initial angiogenesis in vivo. Microvasc. Res..

[113] Ravindran G., Devaraj H. (2012). Aberrant expression of CD133 and musashi-1 in preneoplastic and neoplastic human oral squamous epithelium and their correlation with clinicopathological factors. Head Neck.

[114] Irollo E., Pirozzi G. (2013). CD133: To be or not to be, is this the real question?. Am. J. Transl. Res..

[115] Lee S.H., Hong H.S., Liu Z.X., Kim R.H., Kang M.K., Park N.H., Shin K.H. (2012). TNFα enhances cancer stem cell-like phenotype via Notch-Hes1 activation in oral squamous cell carcinoma cells. Biochem. Biophys. Res. Commun..

[116] Ranganathan P., Weaver K.L., Capobianco A.J. (2011). Notch signalling in solid tumours: A little bit of everything but not all the time. Nat. Rev. Cancer.

[117] Radtke F., MacDonald H.R., Tacchini-Cottier F. (2013). Regulation of innate and adaptive immunity by Notch. Nat. Rev. Immunol..

[118] Wilson A., MacDonald H.R., Radtke F. (2001). Notch 1-deficient common lymphoid precursors adopt a B cell fate in the thymus. J. Exp. Med..

[119] Yashiro-Ohtani Y., He Y., Ohtani T., Jones M.E., Shestova O., Xu L., Fang T.C., Chiang M.Y., Intlekofer A.M., Blacklow S.C. (2009). Pre-TCR signaling inactivates Notch1 transcription by antagonizing E2A. Genes Dev..

[120] Kyoizumi S., Kubo Y., Kajimura J., Yoshida K., Hayashi T., Nakachi K., Moore M.A., van den Brink M.R.M., Kusunoki Y. (2017). Fate Decision Between Group 3 Innate Lymphoid and Conventional NK Cell Lineages by Notch Signaling in Human Circulating Hematopoietic Progenitors. J. Immunol..

[121] Schroeder T., Kohlhof H., Rieber N., Just U. (2003). Notch signaling induces multilineage myeloid differentiation and up-regulates PU.1 expression. J. Immunol..

[122] Cheng P., Nefedova Y., Corzo C.A., Gabrilovich D.I. (2007). Regulation of dendritic-cell differentiation by bone marrow stroma via different Notch ligands. Blood.

[123] Jundt F., Pröbsting K.S., Anagnostopoulos I., Muehlinghaus G., Chatterjee M., Mathas S., Bargou R.C., Manz R., Stein H., Dörken B. (2004). Jagged1-induced Notch signaling drives proliferation of multiple myeloma cells. Blood.

[124] Maniati E., Bossard M., Cook N., Candido J.B., Emami-Shahri N., Nedospasov S.A., Balkwill F.R., Tuveson D.A., Hagemann T. (2011). Crosstalk between the canonical NF-κB and Notch signaling pathways inhibits Pparγ expression and promotes pancreatic cancer progression in mice. J. Clin. Investig..

[125] Bano N., Yadav M., Mohania D., Das B.C. (2018). The role of NF-κB and miRNA in oral cancer and cancer stem cells with or without HPV16 infection. PLoS ONE.

[126] Sun Y., Zhu D., Wang G., Wang D., Zhou H., Liu X., Jiang M., Liao L., Zhou Z., Hu J. (2015). Pro-Inflammatory Cytokine IL-1β Up-Regulates CXC Chemokine Receptor 4 via Notch and ERK Signaling Pathways in Tongue Squamous Cell Carcinoma. PLoS ONE.

[127] Erdogan B., Webb D.J. (2017). Cancer-associated fibroblasts modulate growth factor signaling and extracellular matrix remodeling to regulate tumor metastasis. Biochem. Soc. Trans..

[128] Chan J.S., Tan M.J., Sng M.K., Teo Z., Phua T., Choo C.C., Li L., Zhu P., Tan N.S. (2017). Cancer-associated fibroblasts enact field cancerization by promoting extratumoral oxidative stress. Cell Death Dis..

[129] Li X., Xu Q., Wu Y., Li J., Tang D., Han L., Fan Q. (2014). A CCL2/ROS autoregulation loop is critical for cancer-associated fibroblasts-enhanced tumor growth of oral squamous cell carcinoma. Carcinogenesis.

[130] Shao H., Kong R., Ferrari M.L., Radtke F., Capobianco A.J., Liu Z.-J. (2015). Notch1 Pathway Activity Determines the Regulatory Role of Cancer-Associated Fibroblasts in Melanoma Growth and Invasion. PLoS ONE.

[131] Lim K.P., Cirillo N., Hassona Y., Wei W., Thurlow J.K., Cheong S.C., Pitiyage G., Parkinson E.K., Prime S.S. (2011). Fibroblast gene expression profile reflects the stage of tumour progression in oral squamous cell carcinoma. J. Pathol..

[132] Vsiansky V., Gumulec J., Raudenska M., Masarik M. (2018). Prognostic role of c-Met in head and neck squamous cell cancer tissues: A meta-analysis. Sci. Rep..

[133] Marsh D., Suchak K., Moutasim K.A., Vallath S., Hopper C., Jerjes W., Upile T., Kalavrezos N., Violette S.M., Weinreb P.H. (2011). Stromal features are predictive of disease mortality in oral cancer patients. J. Pathol..

[134] Lim Y.C., Han J.H., Kang H.J., Kim Y.S., Lee B.H., Choi E.C., Kim C.H. (2012). Overexpression of c-Met promotes invasion and metastasis of small oral tongue carcinoma. Oral Oncol..

[135] Dong G., Chen Z., Li Z.Y., Yeh N.T., Bancroft C.C., van Waes C. (2001). Hepatocyte growth factor/scatter factor-induced activation of MEK and PI3K signal pathways contributes to expression of proangiogenic cytokines interleukin-8 and vascular endothelial growth factor in head and neck squamous cell carcinoma. Cancer Res..

[136] Knowles L.M., Stabile L.P., Egloff A.M., Rothstein M.E., Thomas S.M., Gubish C.T., Lerner E.C., Seethala R.R., Suzuki S., Quesnelle K.M. (2009). HGF and c-Met participate in paracrine tumorigenic pathways in head and neck squamous cell cancer. Clin. Cancer Res..

[137] Dajee M., Lazarov M., Zhang J.Y., Cai T., Green C.L., Russell A.J., Marinkovich M.P., Tao S., Lin Q., Kubo Y. (2003). NF-kappaB blockade and oncogenic Ras trigger invasive human epidermal neoplasia. Nature.

[138] Nicolas M., Wolfer A., Raj K., Kummer J.A., Mill P., van Noort M., Hui C.C., Clevers H., Dotto G.P., Radtke F. (2003). Notch1 functions as a tumor suppressor in mouse skin. Nat. Genet..

[139] Thélu J., Rossio P., Favier B. (2002). Notch signalling is linked to epidermal cell differentiation level in basal cell carcinoma, psoriasis and wound healing. BMC Dermatol..

[140] Yap L.F., Lee D., Khairuddin A., Pairan M.F., Puspita B., Siar C.H., Paterson I.C. (2015). The opposing roles of NOTCH signalling in head and neck cancer: A mini review. Oral Dis..

[141] Pickering C.R., Zhang J., Yoo S.Y., Bengtsson L., Moorthy S., Neskey D.M., Zhao M., Ortega Alves M.V., Chang K., Drummond J. (2013). Integrative genomic characterization of oral squamous cell carcinoma identifies frequent somatic drivers. Cancer Discov..

[142] Bieging K.T., Mello S.S., Attardi L.D. (2014). Unravelling mechanisms of p53-mediated tumour suppression. Nat. Rev. Cancer.

[143] Beverly L.J., Felsher D.W., Capobianco A.J. (2005). Suppression of p53 by Notch in lymphomagenesis: Implications for initiation and regression. Cancer Res..

[144] Mungamuri S.K., Yang X., Thor A.D., Somasundaram K. (2006). Survival signaling by Notch1: Mammalian target of rapamycin (mTOR)-dependent inhibition of p53. Cancer Res..

[145] Nair P., Somasundaram K., Krishna S. (2003). Activated Notch1 inhibits p53-induced apoptosis and sustains transformation by human papillomavirus type 16 E6 and E7 oncogenes through a PI3K-PKB/Akt-dependent pathway. J. Virol..

[146] Kim S.B., Chae G.W., Lee J., Park J., Tak H., Chung J.H., Park T.G., Ahn J.K., Joe C.O. (2007). Activated Notch1 interacts with p53 to inhibit its phosphorylation and transactivation. Cell Death Differ..

[147] Huang Q., Raya A., DeJesus P., Chao S.H., Quon K.C., Caldwell J.S., Chanda S.K., Izpisua-Belmonte J.C., Schultz P.G. (2004). Identification of p53 regulators by genome-wide functional analysis. Proc. Natl. Acad. Sci. USA.

[148] Qi R., An H., Yu Y., Zhang M., Liu S., Xu H., Guo Z., Cheng T., Cao X. (2003). Notch1 signaling inhibits growth of human hepatocellular carcinoma through induction of cell cycle arrest and apoptosis. Cancer Res..

[149] Henning K., Heering J., Schwanbeck R., Schroeder T., Helmbold H., Schäfer H., Deppert W., Kim E., Just U. (2008). Notch1 activation reduces proliferation in the multipotent hematopoietic progenitor cell line FDCP-mix through a p53-dependent pathway but Notch1 effects on myeloid and erythroid differentiation are independent of p53. Cell Death Differ..

[150] Duan L., Yao J., Wu X., Fan M. (2006). Growth suppression induced by Notch1 activation involves Wnt-beta-catenin down-regulation in human tongue carcinoma cells. Biol. Cell.

[151] Palomero T., Dominguez M., Ferrando A.A. (2008). The role of the PTEN/AKT pathway in NOTCH1-induced leukemia. Cell Cycle.

[152] Chang K.-Y., Tsai S.Y., Chen S.H., Tsou H.H., Yen C.J., Liu K.J., Fang H.L., Wu H.C., Chuang B.F., Chou S.W. (2013). Dissecting the EGFR-PI3K-AKT pathway in oral cancer highlights the role of the EGFR variant III and its clinical relevance. J. Biomed. Sci..

[153] Cai Y., Dodhia S., Su G.H. (2017). Dysregulations in the PI3K pathway and targeted therapies for head and neck squamous cell carcinoma. Oncotarget.

[154] Yang J., Ren X., Zhang L., Li Y., Cheng B., Xia J. (2018). Oridonin inhibits oral cancer growth and PI3K/Akt signaling pathway. Biomed. Pharmacother. Biomed. Pharmacother..

[155] Wong G.W., Knowles G.C., Mak T.W., Ferrando A.A., Zúñiga-Pflücker J.C. (2012). HES1 opposes a PTEN-dependent check on survival, differentiation, and proliferation of TCRβ-selected mouse thymocytes. Blood.

[156] Zheng Y., Wang Z., Ding X., Zhang W., Li G., Liu L., Wu H., Gu W., Wu Y., Song X. (2018). A novel Notch1 missense mutation (C1133Y) in the Abruptex domain exhibits enhanced proliferation and invasion in oral squamous cell carcinoma. Cancer Cell Int..

[157] Rodilla V., Villanueva A., Obrador-Hevia A., Robert-Moreno A., Fernández-Majada V., Grilli A., López-Bigas N., Bellora N., Albà M.M., Torres F. (2009). Jagged1 is the pathological link between Wnt and Notch pathways in colorectal cancer. Proc. Natl. Acad. Sci. USA.

[158] Gekas C., D’Altri C.G.T., Aligué R., González J., Espinosa L., Bigas A. (2016). β-Catenin is required for T-cell leukemia initiation and MYC transcription downstream of Notch1. Leukemia.

[159] Pannone G., Bufo P., Santoro A., Franco R., Aquino G., Longo F., Botti G., Serpico R., Cafarelli B., Abbruzzese A. (2010). WNT pathway in oral cancer: Epigenetic inactivation of WNT-inhibitors. Oncol. Rep..

[160] Buim M.E.C., Gurgel C.A.S., Ramos E.A.G., Lourenço S.V., Soares F.A. (2011). Activation of sonic hedgehog signaling in oral squamous cell carcinomas: A preliminary study. Hum. Pathol..

[161] Wang Y.-F., Chang C.J., Lin C.P., Chang S.Y., Chu P.Y., Tai S.K., Li W.Y., Chao K.S., Chen Y.J. (2012). Expression of hedgehog signaling molecules as a prognostic indicator of oral squamous cell carcinoma. Head Neck.

[162] Schneider S., Thurnher D., Kloimstein P., Leitner V., Petzelbauer P., Pammer J., Brunner M., Erovic B.M. (2011). Expression of the Sonic hedgehog pathway in squamous cell carcinoma of the skin and the mucosa of the head and neck. Head Neck.

[163] Srinath S., Iyengar A.R., Mysorekar V. (2016). Sonic hedgehog in oral squamous cell carcinoma: An immunohistochemical study. J. Oral Maxillofac. Pathol. JOMFP.

[164] Stepan V., Ramamoorthy S., Nitsche H., Zavros Y., Merchant J.L., Todisco A. (2005). Regulation and function of the sonic hedgehog signal transduction pathway in isolated gastric parietal cells. J. Biol. Chem..

[165] Tanaka T., Atsumi N., Nakamura N., Yanai H., Komai Y., Omachi T., Tanaka K., Ishigaki K., Saiga K., Ohsugi H. (2016). Bmi1-positive cells in the lingual epithelium could serve as cancer stem cells in tongue cancer. Sci. Rep..

[166] Rich A.M., Reade P.C. (2001). Epithelial-mesenchymal interactions in experimental oral mucosal carcinogenesis. J. Oral Pathol. Med..

[167] Chang N.W., Tsai M.H., Lin C., Hsu H.T., Chu P.Y., Yeh C.M., Chiu C.F., Yeh K.T. (2011). Fenofibrate exhibits a high potential to suppress the formation of squamous cell carcinoma in an oral-specific 4-nitroquinoline 1-oxide/arecoline mouse model. Biochim. Biophys. Acta.

[168] Zhou G., Hasina R., Wroblewski K., Mankame T.P., Doçi C.L., Lingen M.W. (2010). Dual inhibition of vascular endothelial growth factor receptor and epidermal growth factor receptor is an effective chemopreventive strategy in the mouse 4-NQO model of oral carcinogenesis. Cancer Prev. Res..

[169] Cancer Genome Atlas Network (2015). Comprehensive genomic characterization of head and neck squamous cell carcinomas. Nature.

[170] Nakagawa H., Wang T.C., Zukerberg L., Odze R., Togawa K., May G.H., Wilson J., Rustgi A.K. (1997). The targeting of the cyclin D1 oncogene by an Epstein-Barr virus promoter in transgenic mice causes dysplasia in the tongue, esophagus and forestomach. Oncogene.

[171] Song A.J., Palmiter R.D. (2018). Detecting and Avoiding Problems When Using the Cre-lox System. Trends Genet..

[172] Bian Y., Hall B., Sun Z.J., Molinolo A., Chen W., Gutkind J.S., Waes C.V., Kulkarni A.B. (2012). Loss of TGF-β signaling and PTEN promotes head and neck squamous cell carcinoma through cellular senescence evasion and cancer-related inflammation. Oncogene.

[173] O’Hagan R.C., Heyer J. (2011). KRAS Mouse Models: Modeling Cancer Harboring KRAS Mutations. Genes Cancer.

[174] Caulin C., Nguyen T., Longley M.A., Zhou Z., Wang X.-J., Roop D.R. (2004). Inducible activation of oncogenic K-ras results in tumor formation in the oral cavity. Cancer Res..

[175] Al-Hajj M., Wicha M.S., Benito-Hernandez A., Morrison S.J., Clarke M.F. (2003). Prospective identification of tumorigenic breast cancer cells. Proc. Natl. Acad. Sci. USA.

[176] Rizzo P., Osipo C., Foreman K., Golde T., Osborne B., Miele L. (2008). Rational targeting of Notch signaling in cancer. Oncogene.

[177] Dorai T., Aggarwal B.B. (2004). Role of chemopreventive agents in cancer therapy. Cancer Lett..

[178] Koprowski S., Sokolowski K., Kunnimalaiyaan S., Gamblin T.C., Kunnimalaiyaan M. (2015). Curcumin-mediated regulation of Notch1/hairy and enhancer of split-1/survivin: Molecular targeting in cholangiocarcinoma. J. Surg. Res..

[179] Cecchinato V., Chiaramonte R., Nizzardo M., Cristofaro B., Basile A., Sherbet G.V., Comi P. (2007). Resveratrol-induced apoptosis in human T-cell acute lymphoblastic leukaemia MOLT-4 cells. Biochem. Pharmacol..

[180] Sun Z., Zhou C., Liu F., Zhang W., Chen J., Pan Y., Ma L., Liu Q., Du Y., Yang J., Wang Q. (2018). Inhibition of breast cancer cell survival by Xanthohumol via modulation of the Notch signaling pathway in vivo and in vitro. Oncol. Lett..

[181] Zhang Q., Yuan Y., Cui J., Xiao T., Jiang D. (2016). Paeoniflorin inhibits proliferation and invasion of breast cancer cells through suppressing Notch-1 signaling pathway. Biomed. Pharmacother. Biomed. Pharmacother..

[182] Kiesel V.A., Stan S.D. (2017). Diallyl trisulfide, a chemopreventive agent from Allium vegetables, inhibits alpha-secretases in breast cancer cells. Biochem. Biophys. Res. Commun..

[183] Su G., Chen H., Sun X. (2018). Baicalein suppresses non small cell lung cancer cell proliferation, invasion and Notch signaling pathway. Cancer Biomark. Sect. Dis. Mark..

[184] Amin A.R.M.R., Karpowicz P.A., Carey T.E., Arbiser J., Nahta R., Chen Z.G., Dong J.T., Kucuk O., Khan G.N., Huang G.S., Mi S., Lee H.Y., Reichrath J., Honoki K., Georgakilas A.G. (2015). Evasion of anti-growth signaling: A key step in tumorigenesis and potential target for treatment and prophylaxis by natural compounds. Semin. Cancer Biol..

[185] Koduru S., Kumar R., Srinivasan S., Evers M.B., Damodaran C. (2010). Notch-1 inhibition by Withaferin-A: A therapeutic target against colon carcinogenesis. Mol. Cancer Ther..

[186] Sagiv E., Rozovski U., Kazanov D., Liberman E., Arber N. (2007). Gene expression analysis proposes alternative pathways for the mechanism by which celecoxib selectively inhibits the growth of transformed but not normal enterocytes. Clin. Cancer Res..

[187] Shehzad A., Lee Y.S. (2013). Molecular mechanisms of curcumin action: Signal transduction. BioFactors Oxf. Engl..

[188] Singh A.K., Sharma N., Ghosh M., Park Y.H., Jeong D.K. (2017). Emerging importance of dietary phytochemicals in fight against cancer: Role in targeting cancer stem cells. Crit. Rev. Food Sci. Nutr..

